# Development of a Self‐Deploying Extra‐Aortic Compression Device for Medium‐Term Hemodynamic Stabilization: A Feasibility Study

**DOI:** 10.1002/advs.202412120

**Published:** 2024-12-27

**Authors:** Adrienne Ji, James Davies, Phuoc Thien Phan, Chi Cong Nguyen, Bibhu Sharma, Kefan Zhu, Emanuele Nicotra, Jingjing Wan, Hoang‐Phuong Phan, Christopher Hayward, Nigel H. Lovell, Thanh Nho Do

**Affiliations:** ^1^ Graduate School of Biomedical Engineering, Faculty of Engineering, and Tyree Institute of Health Engineering (IHealthE) UNSW Sydney Kensington Campus Sydney NSW 2052 Australia; ^2^ School of Mechanical and Manufacturing Engineering Faculty of Engineering UNSW Sydney Kensington Campus Sydney NSW 2052 Australia; ^3^ Department of Cardiology St Vincent's Hospital Sydney NSW 2010 Australia; ^4^ St Vincent's Clinical School Faculty of Medicine UNSW Sydney NSW 2052 Australia

**Keywords:** biorobotics, cardiac assistive device, extra‐aortic conterpulsation, hemodynamic stabilization, self‐deployable soft robotic sleeve, soft robotics

## Abstract

Hemodynamic stabilization is crucial in managing acute cardiac events, where compromised blood flow can lead to severe complications and increased mortality. Conditions like decompensated heart failure (HF) and cardiogenic shock require rapid and effective hemodynamic support. Current mechanical assistive devices, such as intra‐aortic balloon pumps (IABP) and extracorporeal membrane oxygenation (ECMO), offer temporary stabilization but are limited to short‐term use due to risks associated with prolonged blood contact. This research presents a novel proof‐of‐concept soft robotic device designed with the aim of achieving low‐risk, medium‐term counterpulsation therapy. The device employs a nature‐inspired growing mechanism for potentially minimally invasive deployment around the ascending aorta, coupled with hydraulic artificial muscles for aortic compression. It demonstrated a maximum stroke volume of 16.48 ± 0.21 mL (SD, n = 5), outperforming all other non‐pneumatic extra‐aortic devices. In addition, in vitro tests with a mock circulation loop (MCL) show a drop in aortic end‐diastolic pressure by 6.32 mmHg and enhance coronary flow under mild aortic stenosis, which attenuate the device's assistive effect. These findings highlight the device's strong potential for optimization as a promising solution to improve outcomes for hemodynamically unstable HF patients.

## Graphical abstract

A proof‐of‐concept device designed for minimally invasive, medium‐term hemodynamic support is developed. Utilizing a growing mechanism, the device autonomously wraps around the aorta and employs soft artificial muscles to deliver extra‐aortic compression. Preliminary in vitro results suggest its potential to enhance cardiac output and reduce ventricular workload, offering a promising solution for patients with hemodynamic instabilities.

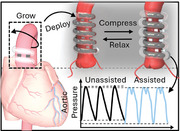

## 1 Introduction

**Figure 1:**
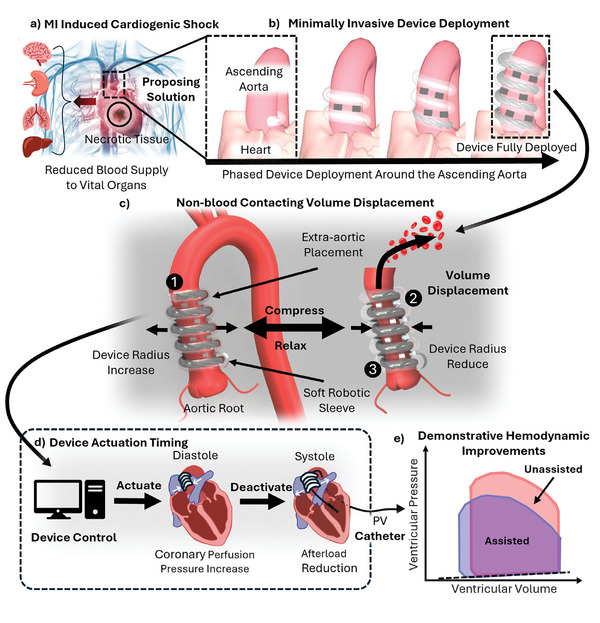
Overview of the device design concept and its working principle. a) Illustration of cardiogenic shock caused by myocardial infarction (MI), resulting in reduced blood supply to vital organs such as the brain, kidneys, lungs, and liver. b) Phased deployment of the proposed device around the ascending aorta. c) Demonstration of the device's volume displacement capability through external compression and relaxation of the aorta. The device consists of 1) an outer soft tubular growing skin, 2) a stabilization mechanism, and 3) internal soft artificial muscles to support its function. d) Operation timing of the device, synchronized with the diastole and systole phases of the cardiac cycle to improve coronary perfusion and reduce afterload. e) Illustration of potential hemodynamic improvements in ventricular pressure‐volume relationship with and without device assistance.

The heart is the central organ of the circulatory system, responsible for pumping oxygenated blood throughout the body to ensure that all organs and tissues receive the necessary nutrients and oxygen to function properly. Its efficient operation is crucial for maintaining homeostasis and overall health. However, several acute and chronic cardiac conditions, such as hypertension, acute coronary syndrome, and valvular heart disease, can impair the heart's ability to pump blood effectively.^[^ ^1^ ^]^ These conditions often result in hemodynamic abnormalities such as changes in blood pressure, stroke volume, and flow, leading to compromised oxygen delivery to tissues. If these abnormalities are not managed appropriately, they can progress to heart failure (HF), a condition where the heart is unable to meet the body's circulatory demands.^[^ ^2^ ^]^ This failure to maintain the balance between oxygen demand and supply can lead to end‐organ damage, particularly affecting vital organs such as the lungs and kidneys, significantly increasing morbidity and mortality (**Figure** **1**a).^[^ ^3^ ^]^

In instances of acute cardiac events, such as myocardial infarction, which can lead to cardiogenic shock in severe cases, the heart may abruptly lose its ability to sustain adequate blood flow. Therefore, achieving hemodynamic stabilization becomes a critical component of medical management. This involves the use of mechanical circulatory support(MCS) devices to reestablish sufficient blood pressure and cardiac output.^[^ ^4^ ^]^ The primary objective is to prevent end‐organ ischemia and subsequent oxygen debt, thereby reducing the risk of further systemic complications.^[^ ^5^ ^]^ Such stabilization provides a temporal window for myocardial recovery and enables the strategic planning and execution of additional therapeutic interventions, ultimately enhancing survival rates and clinical outcomes.

A range of MCS devices are designed to target specific hemodynamic abnormalities and provide varying durations of support depending on the clinical scenario. Hypotension, characterized by reduced systolic and diastolic pressures due to lower cardiac output, often accompanied by a narrowed pulse pressure, is a key feature of cardiogenic shock.^[^ ^3^ ^]^ This reduction in blood pressure leads to compromised perfusion, increasing the risk of organ dysfunction. In such cases, aortic blood pressure can drop significantly from the normal range of 90–120 mmHg, depending on the extent of cardiac compromise. To counteract this hemodynamic instability, counterpulsation devices such as the intra‐aortic balloon pump (IABP) are particularly effective. These devices synchronize with the cardiac cycle, increasing aortic pressure during diastole to enhance coronary and systemic perfusion while reducing afterload during systole to improve cardiac output and decrease myocardial oxygen demand. Aortic assistive devices alike are especially useful in managing severe cardiac conditions where immediate, minimally invasive hemodynamic support is required. In addition to cardiogenic shock, they are also used in cases of acute decompensated HF and post‐cardiac surgery recovery to stabilize patients and prevent further complications.^[^ ^6^ ^]^

However, the IABP, the most widely used counterpulsation device, is typically limited to short‐term use, ranging from several hours to a few days.^[^ ^7^, ^8^ ^]^ This short duration offers minimal opportunity for tissue recovery from oxygen debt and compensatory mechanisms, making it insufficient for sustained hemodynamic stabilization.^[^ ^5^ ^]^ Although devices like Impella and extracorporeal membrane oxygenation(ECMO) provide longer durations of support compared to the IABP, they come with increased risks, higher costs, and more invasive implantation.^[^ ^9^, ^10^ ^]^ Their use typically extends from days to weeks, but each additional day raises the risk of complications such as infection, bleeding, or thrombosis.^[^ ^11^, ^12^ ^]^ A low‐risk, medium‐term counterpulsation device is, therefore, crucial to safely bridge the gap between short‐term treatments and more invasive long‐term support. It would offer a safe, effective, and more affordable alternative for patients needing stabilization beyond the short term. By providing sustained hemodynamic support, such a device could improve patient outcomes, reduce the need for multiple interventions, and lower overall healthcare costs by efficiently managing hemodynamic instability.

One of the key factors limiting the use of IABP to short durations is its blood‐contacting interface, which increases the risk of thrombotic events and bloodstream infections.^[^ ^13^ ^]^ Therefore, a medium‐term counterpulsation device would ideally avoid blood contact, enabling low‐risk support that can last for several days to weeks. In response, several extra‐aortic counterpulsation devices are currently under development. Among these emerging technologies, the C‐Pulse (Sunshine Heart Inc., Tustin, CA) showed promise. It employed a polyurethane balloon, driven by an external pneumatic pump, which inflated and deflated around the ascending aorta to assist the heart. Although human trials supported its ability to provide hemodynamic augmentation,^[^ ^14^, ^15^, ^16^ ^]^ the C‐Pulse still faced significant drawbacks, including the bulkiness of its drivers, operational noise, leaks of the encircling balloon, and the complexity of implantation.

Newer solutions seek to diversify actuation mechanisms to address the previously identified challenges commonly associated with pneumatic devices. Emerging technologies explore actuation methods such as magnetic fields,^[^ ^17^ ^]^ dielectric elastomers (DEA),^[^ ^18^ ^]^ electrohydraulic systems (HASEL),^[^ ^19^ ^]^ and shape memory alloys to achieve extra‐aortic compression. However, many of these approaches still struggle to provide large stroke volumes and raise additional safety concerns. A more detailed comparison of these devices is provided in a later section and in Table S1 (Supporting Information). These challenges highlight the ongoing need for innovation to fully address current limitations.

Building on prior works on extravascular and extraventricular MCS devices, where substantial in vivo tests have demonstrated the feasibility of applying mechanical compression to the aorta and ventricles (e.g. C‐Pulse,^[^ ^14^ ^]^ soft robotic sleeves,^[^ ^20^, ^21^, ^22^, ^23^ ^]^ and PediBooster^[^ ^24^ ^]^), we designed an extra‐aortic counterpulsation device (EACD). The device is designed with the goal of providing enhanced hemodynamics while aiming for safe and reliable operation. Initial proof‐of‐concept results for the EACD are promising, supporting the need for further in vivo and durability testing to evaluate its long‐term safety and reliability. The EACD combines hydraulic and pneumatic actuation to achieve its full functionality, with each type chosen to leverage its distinct advantages in line with the device's operational needs. The initial, one‐time deployment uses relatively low‐pressure pneumatic actuation, chosen for its lightweight nature. For ongoing device actuation, hydraulic actuators are used to enhance safety, as its failure would typically lead to harmless leakage, especially when saline is used as the driving medium. Additionally, the hydraulic actuators require a small input volume and offer high efficiency, creating the potential for implantable designs that could improve patient mobility during support.

The proposed EACD features a novel deployment mechanism, utilizing a preformed soft‐growing robot to position hydraulically driven artificial muscles helically around the ascending aorta (Figure 1b). The helical arrangement is necessitated by the deployment method, as other geometries cannot be achieved through growth‐based positioning. This approach suggests the possibility for a minimally invasive implantation process and avoids blood contact, offering distinct advantages over conventional cuff‐type extravascular compression devices. As shown in Figure 1c, the device provides hemodynamic support by alternately shortening and elongating the artificial muscles to compress and relax the aorta, mimicking the working principle of the IABP. The device operates externally around the ascending aorta, with its actuation timing outlined in Figure 1d. To validate its feasibility, an in vitro test using a mock circulation loop (MCL) was conducted. The expected trends, as illustrated in Figure 1e, suggest improvements in cardiac efficiency and coronary flow, both critical for recovery and stabilization. These features demonstrate the device's potential for low‐risk, medium‐term hemodynamic support, particularly in severe cases like cardiogenic shock. This paper introduces the working principle and demonstrates the initial feasibility of a proof‐of‐concept device aimed at improving patient outcomes in HF management.

## 2 Result

### 2.1 Extra‐Aortic Counterpulsation Device(EACD)

#### 2.1.1 Design and Fabrication

**Figure 2:**
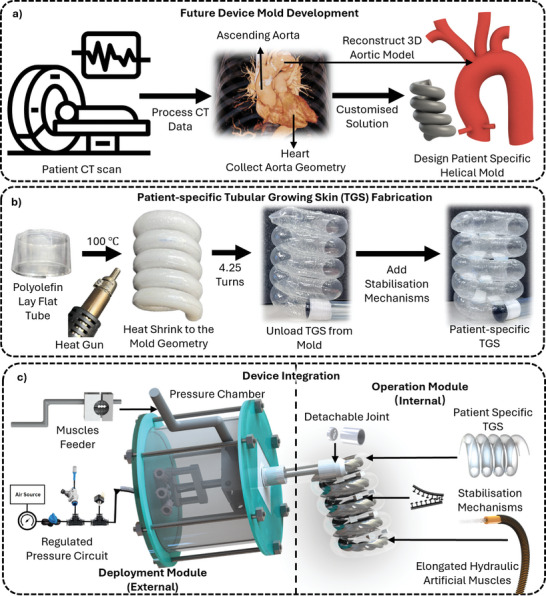
Illustration of the device's fabrication process. a) Planned future mold design for patient‐specific device fabrication. b) Fabrication of TGS using heat‐shrink technology and stabilization mechanisms. c) Integration of the TGS with supporting components into a complete hemodynamic support device, featuring both a deployment and operation module.

The concept of the EACD is illustrated in Figure 1, showcasing its two primary features: 1) an automated deployment mechanism that opens the possibility for minimally invasive placement around the ascending aorta and 2) alternating compression and relaxation of the aorta. **Figure** **2** outlines the fabrication process, involving several critical steps to ensure the device's intended functionality.

The future fabrication process for this device is expected to begin with acquiring patient‐specific geometry of the ascending aorta using computed tomography (CT) scanning (Figure 2a). This step enables device customization to match the patient's anatomy while also identifying and excluding those with high‐risk aortic conditions such as aneurysms or severe dilation. This dual‐purpose approach enhances both the performance and clinical safety of the device. However, in acute cases where time is critical, a one‐size‐fits‐all device would be utilized to ensure rapid deployment. As clinical protocols become more established in the future, further optimization of the fabrication steps is anticipated.

Currently, the device remains in the proof‐of‐concept stage, where patient‐specific data is not yet available. For the purposes of this study, general aortic geometry parameters derived from published literature were used to approximate the aorta's dimensions in a clinically relevant manner.^[^ ^25^ ^]^ These geometrical measurements are utilized to generate an aortic computer‐aided design (CAD) model, which sets the parameters for the creation of a helical mold. This mold is used to fabricate the tubular growing skin (TGS), which forms the device's outer layer and facilitates deployment. The TGS functions as a growing robot, extending its length under pressure via tip eversion.^[^ ^26^ ^]^ To create the TGS, a 0.06 mm thick Polyolefin (POF) layflat tube is heat‐shrunk around the helical mold at 100°C, conforming it to the desired shape. Pre‐trimmed 10 × 10 mm Velcro and dual lock fasteners are then alternately attached to the TGS using an adhesive bond to maintain the structural integrity of the TGS's helical shape, ensuring it retains its form even after pressure is withdrawn (Figure 2b). The hermeticity of the TGS is tested at this stage, ensuring its ability to build the necessary internal pressure for the subsequent deployment phase.

Next, the soft hydraulic artificial muscles are fabricated. The artificial muscles, which generate the compressive forces essential to the device's function, are fabricated following the process detailed in recent work,^[^ ^27^, ^28^ ^]^ and as such are not further illustrated here. Briefly, this involves inserting a rubber tube into a spring coil of similar diameter and binding them together using mechanical bonds and adhesives. One end of the artificial muscles is connected to a fluid transmission line, which links to a fluid reservoir. When pressurized, the radial constraints imposed by the spring cause the muscle to elongate axially, and together with the stabilization mechanism, this enables the compression of the aorta. Each muscle has a 3.18 mm diameter, specifically chosen to prevent radial expansion and minimize environmental interaction. The muscles remain compliant in all states, deforming under external forces to reduce stress on surrounding tissues during operation. After fabrication, the hydraulic artificial muscles are inserted into the TGS and mechanically assembled for deployment.

The final assembly of the device is then completed, integrating all the key components for proper functionality. As shown in Figure 2c, several supporting elements are crucial to the device's functionality. The feeder mechanism enables precise extrusion of the hydraulic muscles into the helically growing TGS, allowing controlled growth during deployment. Meanwhile, the pressure chamber regulates the pressure necessary for driving the TGS's extension. Further fabrication details of these components are provided in Section S1 (Supporting Information). The fully assembled system is divided into two modules: 1) the external deployment module, which is removed after successful deployment, and 2) the internal operation module, which remains within the body to provide long‐term hemodynamic support (Figure 2c).

#### 2.1.2 Device Working Principle

A detailed implantation guide for the EACD is provided in Figure S1 (Supporting Information) to illustrate the device setup. The implantation steps described here are preliminary and provide an example of the suggested methodology for device deployment. These steps focus primarily on the technical aspects of device deployment and are subject to refinement through future in vivo experimental studies. The surgical procedure itself requires further optimization and definition. In its initial state, the device includes a fully inverted TGS joined to artificial muscles, all housed within the pressure chamber. As pressure is increased, the TGS extends via tip eversion. The artificial muscles are fed forward using a hand‐crank‐controlled feeder, allowing the device to deploy in a controlled manner around the ascending aorta(Figure 1b). As growth progresses, the pre‐aligned stabilization mechanism pairs engage and lock the device in place to ensure secure positioning around the aorta. Once deployment is completed and growth‐supporting components are removed, the elongated artificial muscles are connected to external linear actuators. These actuators control the contraction and elongation of the muscles by regulating hydraulic pressure, which in turn controls the compression and relaxation of the aorta(Figure 1c).

The device's operation is synchronized with the cardiac cycle, employing a counterpulsation mechanism to support patients with hypotensive conditions, as shown in Figure 1d. During diastole, immediately after the aortic valve closes, the device applies controlled external compression to the aorta, raising diastolic aortic pressure and mean arterial pressure (MAP). This increase enhances end‐organ perfusion, including the myocardium, by promoting coronary blood flow through a greater pressure gradient at the aortic root. Improved myocardial perfusion delivers oxygen‐rich blood when the heart is most receptive, helping to stabilize cardiac function and support recovery from acute damage.

As the heart transitions to systole, the device relaxes just before the aortic valve opens, lowering aortic root pressure and reducing left ventricular afterload. This temporary vacuum effect decreases the resistance the ventricle must overcome to eject blood, reducing myocardial oxygen demand and energy expenditure. By minimizing wall stress, the device improves myocardial efficiency and facilitates blood ejection with reduced effort.

This coordinated sequence of diastolic augmentation and systolic unloading optimizes hemodynamics. By leveraging the counterpulsation principle, the EACD enhances coronary perfusion during diastole and reduces ventricular workload during systole, promoting myocardial recovery. Counterpulsation therapy prioritizes reducing myocardial strain over directly increasing cardiac output. By lowering ventricular workload, it allows the heart to function more efficiently. In cases of impaired cardiac function, this reduction supports myocardial stabilization and recovery, indirectly contributing to improved hemodynamics.

### 2.2 Modular Testing of the EACD Components

#### 2.2.1 Deployment Mechanism

The TGS is a critical component for the automated deployment of the device. This structure is inspired by a natural growth process, where the component extends its length along a predetermined helical path through tip eversion.^[^ ^26^, ^29^, ^30^ ^]^ This unique mechanism allows the TGS to navigate complex and confined spaces, making it highly suitable for the delivery of artificial muscles in the EACD.

##### Growth Pressure

**Figure 3:**
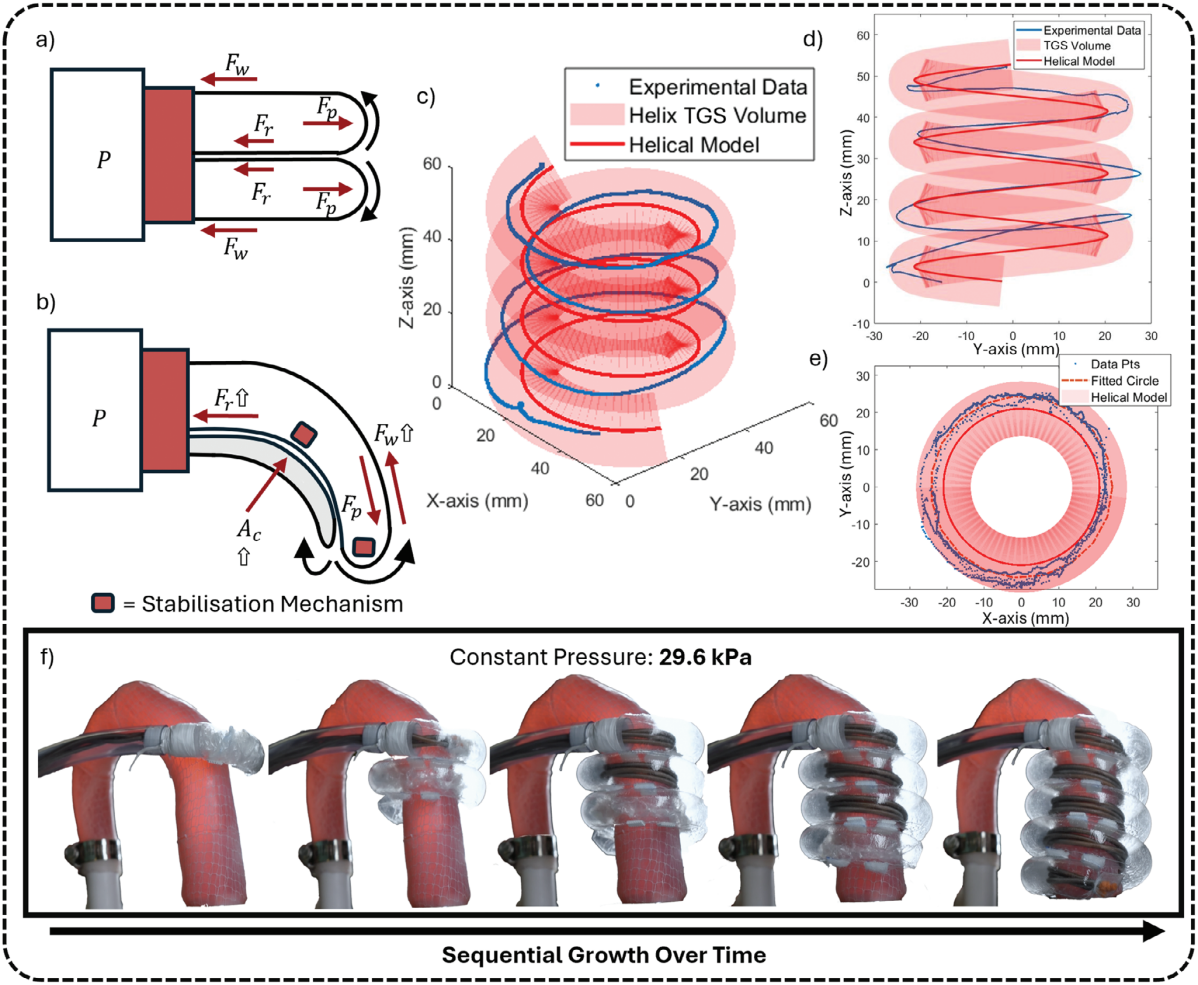
Working Principle and Characterization of the TGS. a) Free body diagram of a straight‐path TGS during growth. b) Free body diagram of high‐curvature preformed TGS during growth. c,d,e) Reconstructed magnetic tracking data of the TGS's growth path from various perspectives (isometric, side, and top views respectively). g) Time‐lapse sequence showing the growth progression of the TGS under constant pressure, deploying artificial muscles around the phantom aorta.

As illustrated in **Figure** **3**a, the working mechanism of the growth of TGS involves several key forces,^[^ ^31^ ^]^ consisting of the forward propelling force (*F*_*p*_) generated as internal pressure builds up within the TGS body, the resistive force (*F*_*w*_) due to frictional interaction with its environment and an internal friction force (*F*_*r*_) between the everted TGS and its internal wall. Growth occurs when the forward propelling force (*F*_*p*_) exceeds the sum of the frictional force (*F*_*w*_) and the internal resistive force (*F*_*r*_):

(1) $$F_{p}>F_{w}+F_{r}$$

The magnitude of these force components is influenced by several variables. The propelling force *F*_*p*_ is generated by internal pressure and can be mathematically expressed as *F*_*p*_ = *PA*, where *P* is the internal pressure and *A* is the cross‐sectional area at the tip of the TGS. This equation indicates that a reduction in the cross‐sectional area requires a proportional increase in pressure to maintain the same propelling force, highlighting the challenge of growing small‐diameter tubular skins. Since the pressure a TGS can withstand is always finite, a higher pressure requirement increases the risk of rupture, which could lead to an energetic explosion and pose safety risks.

The resistive force *F*_*r*_ is an inherent part of the system, since tip eversion of the TGS necessitates relative movement between these two sections. However, it is heavily influenced by the geometry of the TGS. For complex geometries, such as a helix, *F*_*r*_ is significantly greater. Figure 3b shows the free‐body diagram of a curved path TGS. The contact area between interior of TGS and everted material has increased significantly compared to the straight path TGS shown in Figure 3a. Meanwhile, the required larger pressure to overcome the drag exerts a larger normal force on the contacting areas, leading to a substantial drag being produced. The resistive force can be described by the equation *F*_*r*_ = µ*PA*_*c*_, where µ is the coefficient of friction, *P* is the internal pressure, and *A*_*c*_ is the contact area between the everted TGS and the interior walls.

Lastly, *F*_*w*_, the environmental resistance, plays a crucial role in determining the TGS's growth efficiency. Unlike *F*_*p*_ and *F*_*r*_, which are influenced by the TGS's internal dynamics and geometry, *F*_*w*_ is entirely dependent on the external environment. For example, if the TGS is required to navigate through a highly restricted gap, *F*_*w*_ increases significantly, demanding higher internal pressure to compensate for the reduced net force acting at the tip. This resistance becomes particularly relevant in complex or confined environments, where the TGS's ability to advance can be severely compromised by environmental constraints.

This given relationship highlights that the current application, which requires the TGS to have a small cross‐sectional area (due to the constraint of ascending aorta length) and grow into a helical path, results in a relatively large growth pressure compared to values reported in the literature.^[^ ^30^ ^]^ The current baseline growing pressure of the TGS is around 27.2 ± 3.1 *kPa* (SD, n = 4). Variations in the behavior of each TGS are due to differences introduced during manufacturing, as indicated by the standard deviation in the pressure measurements. When the stabilization mechanism is attached to the TGS, *F*_*w*_ increases due to the added rigidity and surface irregularities of the attached mechanism, which impedes the eversion at the tip. This is evident through the elevation in the growth pressure to 30.3 ± 1.2 *kPa* and can vary based on the exact type and size of the stabilization mechanism used. The TGS is also characterized by its burst pressure, which is 70.1 ± 2.5 *kPa*. The current measurements provide the device with a safety factor of ≈ 2. To further enhance safety, strategies such as applying lubricants or using feeder mechanisms to reduce drag and decrease the force *F*_*r*_ can be employed, leading to a more reliable and safer device.

As a component of a medical device, controlled growth of the TGS is critical. If the tip of the everted TGS is untethered, abrupt growth may occur once the baseline growth pressure is reached, with larger propelling net forces leading to greater tip acceleration–an undesirable outcome in medical applications. To prevent this and reduce internal resistive forces, a feeder mechanism is used. The feeder controls a tether, such as a string, attached to the TGS's tip, allowing for precise, manual control of the growth rate. The speed of TGS growth is regulated by the feeder's operation through the tension applied to the tether.

##### Geometrical Accuracy

Geometrical accuracy is a critical factor in TGS characterization, as it directly affects the success of the deployment. A high degree of conformity to the preplanned shape ensures that the TGS can accurately deploy artificial muscles around the aorta while properly engaging the stabilization mechanisms. The characterization of the TGS's geometric accuracy not only validates the deployment process but also allows for customization to fit different patient anatomies.

Figure 3c–e demonstrates the growth path of the TGS in comparison to its programmed path from side, top and iso view, fully defining the TGS's growth path. As identified by the top view profile (Figure 3e), the growth path tracking data is fitted with a circle of radius 24 mm, which is 8.58% (≈ 2 mm) larger than the programmed radius of 22 mm. This discrepancy is proven consistent through repetitions, meaning that it can be accounted for as a tolerance during the device design.

The discrepancies result from a combination of TGS deformation under pressure and measurement error. As pressure accumulates in the TGS, its diameter enlarges slightly due to small wrinkles or deformations in the material that expand under pressure. This behavior occurs primarily during the first TGS growth, likely due to the initial settling of the material, while subsequent growths remain consistent. The elasticity of the material also allows the TGS to stretch slightly when radial expansion forces become significant. Measurement errors arise from the setup of the sensor, which has a rigid tip fixed to the TGS's tip. This configuration allows for free sensor movement within the TGS. When tracing a circular path, the sensor bends along the curve, deflecting toward the outer diameter. This deflection was observed during the experiments and is difficult to avoid due to the inherent flexibility of soft robotics, especially at the tip of the TGS during growth. At this everted stage, there is no pressure maintaining the TGS's shape until it is fully grown.

Additionally, as shown in the side profile (Figure 3e), the traced helix appears slightly tilted to the right, suggesting a potential rotation around the x‐axis. This tilt is primarily due to error accumulation during growth, particularly in the first two turns of the helix. The magnetic tracking system only reports the current x, y, z coordinates, meaning each measurement is independent of previous ones. As a result, error accumulates in the physical TGS, but the sensor only captures the latest data, not accounting for prior deviations. This leads to a more pronounced deviation from the baseline helix in the final two turns of TGS growth. Also, as the TGS grows freely in space, it is easy to deviate slightly from its planned path, with these deviations becoming more obvious at higher growth rates, where the TGS's behavior becomes less controlled. If the TGS were guided by a fixed central structure, the growth path would likely be more precise, minimizing the observed tilt and overall deviations.

Overall, the results indicate that the TGS's growth path is largely within the programmed workspace, demonstrating reliable and consistent performance despite small imperfections in the fabrication process. Figure 3f illustrates the TGS's growth, with the stabilization mechanism in place and tether provided by artificial muscles. Growth occurred at a constant pressure of 29.6 *kPa*, with the feeder controlling the speed through the regulated feeding of the muscles outside the frame. It visually confirms the TGS's ability to maintain the programmed shape during growth. This consistency was observed across multiple samples (n > 10), reinforcing the TGS's reliability in achieving the desired preforms necessary for successful deployment. These findings suggest that the TGS is well‐suited for its intended application, providing a stable foundation for the device's operational success.

#### 2.2.2 Contractile Mechanism

**Figure 4:**
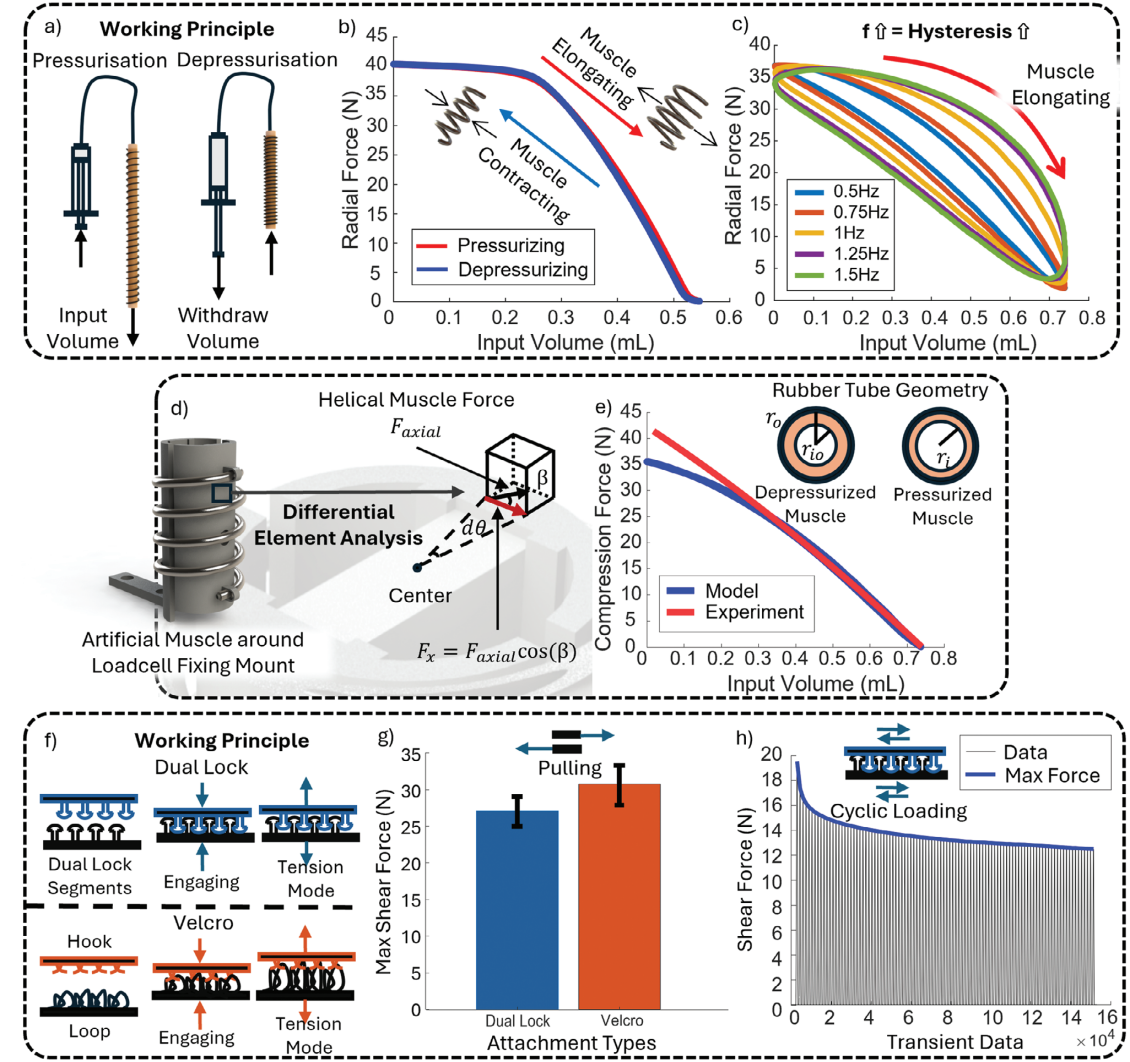
Working Principle and Characterization Test Results for Device Modules. a) Hydraulic artificial muscle working principle. b) Radial force changes during muscle contraction and elongation in a helical configuration under quasistatic conditions. c) Hysteresis behavior of the muscle at different frequencies (0.5 Hz to 1.5 Hz) under quasistatic conditions. d) Free body diagram of a finite element on the load cell structure. e) Comparison of model and experimental radial force data for pressurized and depressurized muscle. f) Stabilization mechanism working principle using dual lock and Velcro attachments. g) Maximum shear force comparison between dual lock and Velcro attachments with the error bar indicating the standard deviations (n = 4) h) Cyclic loading data depicting transient and maximum forces during repeated loading of the stabilization mechanism over 3000 cycles.

The effectiveness of the device in performing counterpulsation therapy is largely dependent on the performance of its contractile elements, which consist of multiple hydraulic artificial muscles for stronger compression force. Thoroughly studying the characteristics of a single artificial muscles is crucial, as it will guide future device optimizations and enable more accurate predictions of the overall device performance. The working principle of the artificial muscle used in the device functions is illustrated in **Figure** **4**a. As internal pressure increases with the addition of input volume, the muscle elongates as the metal coil prevents radial expansion. When the input volume is withdrawn, the internal pressure decreases, allowing the muscle to return to its original length and generate a contraction force.

Figure 4b illustrates the radial force exerted by the muscle during blocked elongation on a fixed‐radius load cell mounting fixture, with only the two ends secured to accurately simulate the muscle's condition within the device. As the artificial muscle is arranged in a helical path, its pressurization and depressurization translate into changes in the helical muscle's radius, as shown in Figure 4b, leading to radial force production. The muscles used in the EACD are 3.18 mm in diameter with an elongated length of 420 mm, designed to loosely wrap around the load cell fixing mount, completing 4 turns. Figure 4b outlines a linear increase in radial force, followed by a plateau with a maximum force production of 38.03 N on a fixed radius (29 mm) load cell mount structure. Beyond this maximum force, the input volume becomes insufficient to maintain the rubber tube's circular shape, leading to its collapse and a loss of contact between the coil and the rubber tube. Consequently, the muscle no longer acts as an entity to exert force but rather as separated components, leading to no significant increase in force. This phenomenon illustrates that at every diameter, the muscle has a maximum force it can produce. For counterpulsation applications, the muscle must exert enough force to deform the aorta and displace significant volume to cause a pressure rise. Given the maximum force production of a single muscle (38.03 N), multiple muscles may be required to achieve the desired therapeutic effect. Since the aorta is a pressurized artery, deforming it requires significant force, likely exceeding what a single muscle can produce. Using multiple muscles in tandem allows for a combined force that can reach the levels necessary to deform the aorta and displace the volume required to generate a pressure rise. It is crucial to operate within each muscle's effective range, defined as the linear region before force production plateaus, to ensure maximum muscle efficiency.

Furthermore, Figure 4b demonstrates a linear relationship between input volume and output radial force, a characteristic attributed to the incompressibility of the hydraulic medium. This relationship simplifies device modeling and control, providing a distinct advantage over pneumatic muscles like McKibben muscles where the muscle is stiffened under applied pressure. The compressibility of air varies with factors such as temperature, humidity, and other environmental conditions, leading to significant and unpredictable hysteresis in muscle response and adding complexity to modeling efforts.^[^ ^32^ ^]^ Interestingly, a small initial kick is observed at the beginning of the force increase, likely due to the presence of air bubbles within the muscle. These bubbles compress until the incompressible liquid fully takes over, after which further increases in input volume directly translate into output force. This observation underscores the differences in behavior between muscles‐driven by hydraulic versus pneumatic sources, with hydraulic systems offering more predictable and consistent performance due to the direct translation of input volume into pressure rise.

Lastly, the frequency response of the muscle is critical for its effectiveness as a cardiac assistive device, where rapid and precise actuation is essential. Figure 4c demonstrates that with increasing input frequency, the hysteresis, indicated by the area between the loading and unloading curves, also increases. This rise in hysteresis is largely attributed to the inherent properties of the elastic tube material at larger strains, internal resistance to fluid flow in longer muscles, and friction between the outer constraining coils and the rubber tube^[^ ^33^ ^]^ These factors result in greater energy loss and make achieving precise control more difficult.

The increased hysteresis at higher frequencies poses a challenge for the device's ability to provide effective counterpulsation, as energy loss and reduced control accuracy can limit its performance in supporting rapid cardiac cycles. Strategies to mitigate such hysteresis include applying pre‐tension or pre‐load to the muscle to reduce slack and response delays, using materials with lower viscoelasticity to replace the rubber tube, and optimizing the muscle's geometry to reduce internal resistance. Additionally, optimizing the muscle design to limit strain within the linear elastic region, where the material behaves predictably, would reduce hysteresis and further enhance performance.^[^ ^34^ ^]^ Alternatively, advanced control strategies, such as adaptive modeling and compensation techniques,^[^ ^35^ ^]^ could be implemented to account for the effects of hysteresis and maintain precision.

#### 2.2.3 Empirical modeling of the Radial Force of the EACD

To better understand the performance of the actuator, an empirical model is derived. This model allows for future optimization and the development of control algorithms to ensure reliable operation under various conditions. Given that the contractile elements are hydraulic artificial muscles, whose axial force output has been mathematically derived in previous studies,^[^ ^27^, ^36^ ^]^ we will use these derivations as a foundation to tailor the model specifically for our application. Several assumptions are necessary to derive this axial force, they are outlined in Table S3 (Supporting Information). In previous models, the axial force was a function of muscle displacement*x* and muscle pressure*P*_*m*_. For a more straightforward control, we aim to use the input volume to directly infer the output force. Based on prior work, the axial force of the muscle can be expressed as:

(2) $$F_{out}=E\pi \left(r_{o}^{2}-r_{i}^{2}\right)\left(\frac{\varepsilon }{1+\varepsilon }\right)+K_{c}l_{o}\varepsilon -P_{m}\left(\frac{\pi (\varepsilon r_{o}^{2}-r_{i}^{2})}{1+\varepsilon }\right)$$

where *E* is the experimentally determined elastic modulus of the rubber tube, *r*_*o*_ and *r*_*i*_ represent the outer and inner radii of the rubber tube, respectively, as shown in Figure 4e. *l*_*o*_ denotes the initial length of the muscle, ϵ is the strain of the artificial muscle and *K*_*c*_ is the spring constant of the outer coil. The values and further details of these parameters are provided in Table S4 (Supporting Information).

However, it was previously simplified that the axial force is the only output force from the muscle. The actual output force is a combination of axial force, hoop force, radial force, and torsional force:

(3) $$F_{out}=F_{axial}+F_{hoop}+F_{radial}+F_{torsional}$$

Due to the radial equilibrium assumption, *F*_*radial*_ = 0. Similarly, the hydrostatic assumption ensures *F*_*hoop*_ = 0, as internal pressure acts uniformly along the curved surface, producing a balanced overall hoop force on the structure. Therefore, the equation simplifies to:

(4) $$F_{out}=F_{axial}+F_{torsional}=\lambda F_{out}+\gamma F_{out}$$

where λ and γ represent the coefficients for the axial and torsional forces, respectively, with λ + γ = 1. The value of λ is expected to represent the majority of the force output since the coil's ability to twist during elongation is limited. By comparing the modeled data with experimentally obtained axial force outputs as shown in Figure S2 (Supporting Information), λ has been determined to be 0.87. This adjustment accounts for previously overlooked non‐axial forces, which significantly impact the accuracy of the modeled forces. This is especially relevant when the muscle wraps around a structure with many turns, where accumulated errors can become substantial.

Subsequently, to add the variable input volume *V*_*in*_ into the equation, we use the relationship between *P*_*m*_ and *V*_*in*_. Since the volume contained within the muscle is dependent on the size of the cavity of the rubber tube, *V*_*in*_ can be expressed as $V_{in}=\pi r_{i}^{2}l_{o}\left(1+\varepsilon \right)$.^[^ ^36^ ^]^ By definition, $\varepsilon =\frac{x}{l_{o}}$, thus we can conclude:

(5) $$V_{in}=\pi r_{o}^{2}x$$

Due to the principle of energy conservation, the total mechanical energy of the system can be expressed as *W*_*p*_ = *W*_*c*_ + *W*_*r*_ + *W*_*l*_.^[^ ^37^ ^]^ This equation indicates that the work done by the muscle (*W*_*p*_) is the sum of the work done by the rubber tube (*W*_*r*_) and the coil (*W*_*c*_), plus any energy loss. In this context, we assume perfect efficiency with no energy dissipated as heat or friction (*W*_*l*_ = 0) as the system is idealized for modeling purposes.

The equivalent expanded form of the energy conservation equation gives:

(6) $$P_{m}V_{in}=\frac{1}{2}K_{c}x^{2}+\frac{1}{2}K_{r}x^{2}$$

By substituting the relationship between *V*_*in*_ and displacement *x* into the energy conservation equation, the muscle pressure can be derived as:

(7) $$P_{m}=\frac{V_{in}}{2{\left(\pi r_{o}^{2}\right)}^{2}}\left(K_{c}+K_{r}\right)$$

where

(8) $$K_{r}=\frac{\pi^{2}El_{o}A_{ro}r_{o}^{4}}{{\left(\pi r_{o}^{2}l_{o}+V_{in}\right)}^{2}}$$

*K*_*r*_ is the spring constant of the rubber tube which is a function of *V*_*in*_ and *A*_*ro*_ is the initial cross‐sectional area of the rubber tube.

Thus far, the axial force *F*_*axial*_ of the muscle is converted into a function of *V*_*in*_ where all other terms are constants representing the material properties of components of the muscle. Progressing further, we converted the axial force of the muscle to the radial force using the finite element method. The force a single unit is subjected to on the cylindrical load cell mounting fixture is illustrated in Figure 4d. The helical winding of the muscle results in the force applying to the unit in an oblique direction, making an angle of β with the horizontal. Since only the horizontal component of the force contributes to the generation of radial force, the effective magnitude of the force is:

(9) $$F_{x}=F_{axial}\cos\left(\beta \right)$$

Moving forward, we now derive the compression force caused by the wrapping of the muscle. Previously, we analyzed the forces acting on a unit segment of the load cell mounting fixture that subtends an infinitesimal angle *d*θ at the center, as depicted in Figure 4d. The normal reaction force, or the radial force, acting on the unit due to force *F*_*x*_ is given by:

(10) $$F_{rad}=F_{x}\sin\left(\frac{d\theta }{2}\right)$$

(11) $$F_{rad}=F_{axial}\cos\left(\beta \right)\sin\left(\frac{d\theta }{2}\right)$$

The normal force can be expressed using only the *sin* component because when summing the entry and exit force created by the wrapping of the muscle subtended by the angle of *d*θ, the *cos* component of the force always cancels out each other due to their opposite directions. As a result, only the *sin* component of the *F*_*x*_ contributes to the net normal force.

If we sum the radial force for each infinitesimal *d*θ over a finite angle θ, the overall radial force on that segment becomes:

(12) $$F_{rad}=F_{axia}\cos\left(\beta \right)\int_{0}^{\theta }\sin\left(\frac{\theta }{2}\right)\;d\theta$$

Upon performing the integration over half a cycle, we find that for every half‐revolution, the radial force is:

(13) $$F_{rad}=2F_{axial}\cos\left(\beta \right)$$

We calculate the radial force over a half‐revolution because summing the forces over a full revolution would result in them canceling out due to their opposite directions. However, while the net force over a full revolution is zero, the load cell or underlying structure still experiences compression. Each half‐revolution of the wrapping muscle contributes equally to this compression. Since both halves apply inward forces, the total compression force for a full revolution is the sum of the forces from each half. Consequently, the total radial force for a complete revolution is twice in the magnitude of a single half‐revolution.

Therefore, the radial output force of the muscle with *n* turns can be expressed as:

(14) $$F_{compression}=4\sigma nF_{axial}\cos\left(\beta \right)$$

The variables in the final expression are detailed in **Table** **1** for clarity and reference.

**Table 1:** Table of variables and their definitions as used in the final formula.

Variable	Description
σ	Accounts for force loss due to the friction between the muscle and loadcell structure
*F*_*axial*_	This is a function of *V*_*in*_, its fully expanded form is represented at Equations 7 and 8
*n*	The number of turns the muscle winds around the loadcell structure
*cos*(β)	A constant that can be calculated using the definition $\beta =\arctan(\frac{Helix\;Pitch}{Helix\;Diameter})$.

The model outputs a radial force prediction, as illustrated in Figure 4e, showing strong agreement with the experimental results, particularly in the linear region. While the model has limitations, such as its inability to capture hysteresis and the force plateauing observed at small input volumes, it accurately predicts force within the muscle's working range. This accuracy in the linear region is crucial, as it directly informs the performance of the device during counterpulsation. The ability to reliably predict force in this range ensures that the model can be effectively integrated into the control algorithms for the device, as long as operations are maintained within the muscle's linear range. This will enhance the device's precision and efficiency, especially during critical phases of cardiac support, where maintaining consistent and predictable force output is essential for effective volume displacement.

#### 2.2.4 Stabilization Mechanism

The stabilization mechanism is crucial for the device's operation, as it maintains the muscles in their helical shape after pressure is withdrawn from the TGS. During deployment, the aligned stabilization mechanism pairs engage upon contact to establish an initial lock. Once the pressure is withdrawn, these locking pairs are further reinforced using surgical tools, ensuring the device is securely fixed in place. This ensures the efficient conversion of linear muscle contraction into radial constriction, which is essential for achieving the desired therapeutic effect. Given the need for the stabilization mechanism to be reversible, strong, robust, and safe within a biological environment, traditional attachment methods, such as adhesives or sutures, are unsuitable. Instead, hook‐and‐loop fasteners and dual‐lock mechanisms present unique advantages for this application. Their ability to form a secure yet detachable connection, along with their durability and compatibility with various materials, make them ideal choices. The working principle of these two attachment mechanisms is illustrated in Figure 4f. Upon compression, both systems establish reliable mechanical engagement, allowing them to withstand significant force.

We characterized the stabilization mechanism based on its maximum shear force, as this is the primary direction of force during device operation. To ensure minimal impedance to TGS growth, the stabilization mechanism is trimmed to in small segments (10 × 10 mm). When only a single pair of stabilization patches is used on the TGS, the applied force causes the TGS to rotate, shifting the force direction from shear to tensile. However, by placing two patches at a distance from each other along the TGS, the rotation is restricted as it requires more free space, which is not available within the system. This setup effectively maximizes the force in the shear direction, ensuring that the mechanism operates optimally under these conditions. Both the hook‐and‐loop fasteners and the dual‐lock mechanisms exhibit significantly greater resistance to shear forces compared to tensile forces, ensuring they can reliably maintain the muscle's configuration during operation.

The tests were performed on 10mm × 10mm pieces for both the hook‐and‐loop and dual‐lock mechanisms, same as the size of one pair of stabilization mechanisms used in the device. Figure 4g illustrates that Velcro (hook and loop) can sustain a shear force of 30.75 ± 2.62 N (SD, n = 4) before failure while dual lock holds 27.07 ± 2.01 N before losing grip. Given the small surface area of these mechanisms, both offer exceptional holding strength. The force each patch can hold is directly proportional to the size of the patch, meaning that, with multiple pairs employed in the device, they can collectively withstand a shear force greater than 200 N. This value is roughly five times the maximum force a single muscle is capable of producing, ensuring that the stabilization mechanisms provide the necessary reliability during device operation.

The two types of stabilization mechanisms, Velcro and Dual Lock, exhibit distinct material properties that influence their suitability for the device. Velcro is renowned for its flexibility and thinness, making it ideal for applications requiring a low‐profile attachment. In contrast, Dual Lock is bulkier and more rigid, with its strength attributed to the robust mushroom head pins and rigid substrate, which ensures an even distribution of force across the pins. Despite these advantages, both mechanisms have inherent limitations based on currently available commercial options. Moreover, when compared to conventional surgical adhesives such as hydrogels and BioGlue, the tested mechanisms demonstrate significantly higher mechanical strength, although they lack the biointegration properties needed for seamless integration with biological tissues.^[^ ^38^ ^]^ For the proposed device, mechanical strength is critical to ensure secure muscle attachment. However, both high mechanical strength and biological safety must be prioritized to guarantee successful and safe device implantation. These limitations highlight future directions for optimizing the stabilization mechanism. Customizing the design could help strike an optimal balance between strength and flexibility, potentially combining the secure attachment strength of Dual Lock with the flexibility and low profile of Velcro. Additionally, using bioinert materials could enhance biological safety, addressing the need for both mechanical integrity and biocompatibility. By focusing on these improvements, future iterations of the stabilization mechanism could better meet the demands of both strength and safety in a medical setting.

Finally, given the promising initial results, we proceeded to investigate the stabilization mechanism's ability to withstand cyclic shear loading. While the cyclic loading tests provide valuable insights, it is important to note that the conditions created by using a linear actuator to produce displacement and forces may differ slightly from the actual force production scenario in the device. Both stabilization mechanisms are not intended to function under conditions involving large displacements, as the device itself is designed to operate with relatively small, controlled movements. Therefore, the results from these tests should be seen as indicative of performance trends rather than definitive outcomes for the device's real‐world operation. Despite Velcro's outstanding performance in the static shear tests, its performance under cyclic loading with displacements was significantly lower. This reduced performance is likely due to premature disengagement of the Velcro segments within the oscillating environment at higher frequencies, where the loops slip out of the hooks. Conversely, Dual Lock performed well under repeated stress, as shown in Figure 4h. A linear actuator reproduced cyclic loading conditions by moving according to a small amplitude (4 mm), high‐frequency (0.75 Hz) sine wave. Initially, a shear force of 19.4 N was observed, but it quickly decayed in subsequent cycles, likely due to the deformation of the foam tape adhesives backing the Dual Lock tape. This initial force reduction could also be due to the deformation of the mushroom‐shaped pins after extensive loading. However, despite the drop in force, the structural integrity of the mushroom head pins remained intact, and their engagement continued effectively. This suggests that while some deformation occurs, it does not compromise the mechanism's overall capacity to withstand force. Instead, a larger displacement may be required to achieve the same force due to structural changes over time. This result suggests that the drop in force should not significantly impair the device's overall function, especially given the small scale of displacement produced by the linear actuator. To further investigate, a follow‐up tensile test was conducted immediately after cyclic loading, which confirmed that the maximum force remained consistent at 25.23 N. This value aligned with previous measurements and fell within the expected standard deviation. The consistency in performance after extensive loading highlights the durability and reliability of the Dual Lock mechanism.

These results indicate that Dual Lock can more competently provide secure attachment under conditions of high‐frequency, large‐displacement oscillation. While this test suggests that Velcro may be less effective under such conditions, it does not nullify its suitability as a stabilization mechanism for the device. Instead, it provides critical information about the specific scenarios in which Velcro performs less reliably, offering valuable insights for future device improvements.

### 2.3 Integration Testing on the EACD Under Physiological Pressure

**Figure 5:**
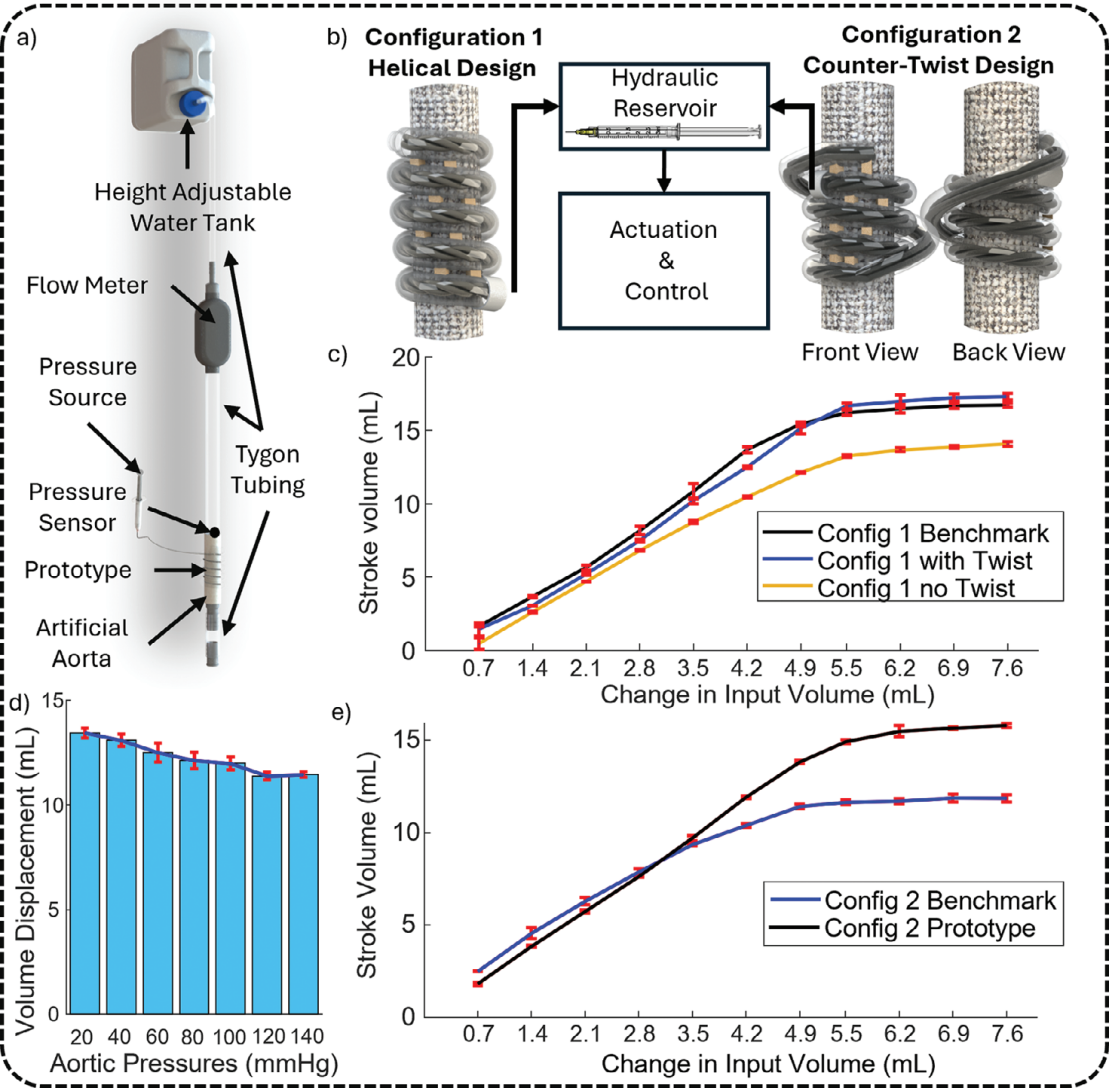
Experimental Setup and Stroke Volume Characterization of Integrated Device. a) Illustrative diagram of the experimental setup. b) An illustration of the two device configurations connected to the linear slider for operation. c) Stroke volume at increasing change in input volume for Configuration 1 with and without twist using rigid and flexible stabilization mechanism. d) Stroke volume at various aortic pressures with constant muscle input volume. e) Stroke volume at increasing change in input volume for Configuration 2 using rigid and flexible stabilization mechanisms.

After studying individual components, the device was assembled and tested under physiological conditions to evaluate its counterpulsation performance using the experimental setup presented in **Figure** **5**a. Its primary function is to displace and reverse the displacement of blood volume under varying pressures, thus modulating arterial pressure. The complete system comprises a TGS equipped with eight pairs of tape segments, which stabilize the hydraulic artificial muscles in their programmed shape during muscle elongation and contraction. To ensure reliable volume displacement at high pressures, three hydraulic artificial muscles are bundled together, collectively applying radial force to the underlying aorta. These muscles are all designed to reach 100% elongation in its most relaxed state which loosely wraps around the ascending aorta to ensure maximal aortic relaxation. Upon receiving a signal, the muscles shorten, compressing the aorta and displacing blood volume effectively.

Figure 5b presents an illustrative comparison of the device in two configurations: Configuration 1: Helical Design and Configuration 2: Counter‐Twist Design. A photograph of the real device in each configuration can be found in Figure S3 (Supporting Information), with a demonstration video provided in Video S2 (Supporting Information). The helical design was selected for its simplicity in deployment, allowing for efficient wrapping around the aorta. However, during testing, we observed that muscle contraction in this configuration caused the underlying aorta to twist. As the muscle shortens, it applies a spiral force, creating torsional stress along with the desired compressive force. Although this twisting effect aids volume displacement, it increases the risk of aortic damage due to torsional forces.

To address this, we introduced Configuration 2: Counter‐Twist Design, where the muscle structure reconnects its tip to its base, creating a counter‐helix that balances forces generated during muscle contraction. This balanced configuration minimizes directional imbalances, ensuring that muscle shortening produces a compressive force without twisting or lateral motion. By eliminating torsional stress, the counter‐twist design enhances device safety and stability during aortic compression. Both configurations were evaluated for volume displacement performance, allowing a direct comparison of their effectiveness.

The device generates volume displacement by shortening the elongated artificial muscle filaments, as shown in Video S3 (Supporting Information). As outlined in the contractile mechanism section, the artificial muscle elongates when pressurized and contracts upon depressurization (Figure 4a). The fully extended state of the muscle is defined as the reference point, where the artificial muscle loosely wraps around the ascending aorta without applying stress or deforming the underlying structure, corresponding to zero change in input volume in Figure 5c,d. Any decrease in input volume from this reference point, expressed as the change in input volume, shortens the muscle, which in turn compresses the aorta and facilitates volume displacement.

Upon analyzing the volume displacement results presented in Figure 5c,e, we observe that both configurations demonstrate consistent stroke volume, with standard deviations remaining below 10% across all input volumes. This consistency confirms the device's ability to function reliably, whether secured with rigid or flexible fixation methods. As a result, further analysis comparing benchmark results from rigid fixations with prototype results from flexible fixations is valid. An exemplar flow profile output by the device is shown in Figure S4 (Supporting Information).

In both configurations, the benchmark volume displacement achieved using rigid fixation methods (black line) consistently exceeds that produced with flexible fixations (blue line), particularly at the higher changes in input volumes. After the volume displacement has plateaued, Configuration 1 shows a consistent volume displacement reduction of 3.03 mL with flexible fixations compared to rigid counterparts, while Configuration 2 exhibits a reduction of 3.25 mL. This reduction in performance occurs because some of the muscle movement is absorbed by the flexible fixation mechanisms, resulting in wasted motion. Rigid fixations ensure that all muscle contraction is directly translated into aortic compression, while flexible fixations dissipate a portion of the muscle shortening into relative motion between the device's turns. This loss is an inherent characteristic of soft robotics, where flexibility comes at the cost of performance efficiency. As the device stroke volume increases, the performance gap becomes more pronounced, with muscle shortening placing greater demands on the stabilization mechanisms, leading to increased strain and relative movement.

However, a few exceptions are observed, particularly at smaller withdrawn input volumes in Configuration 2, where the volume displaced by flexible fixation methods exceeds the benchmark results. This occurs because with flexible fixation spacing between the muscles are increased by the presence of TGS, causing a greater distance between the turns of the device. Subsequently, this enlarges the active surface area of the device, which in turn results in a larger stroke volume. At lower withdrawn volumes, this effect leads to more efficient volume displacement. However, as the withdrawn volume increases, the strain on the flexible fixation mechanisms also increases, leading to a loss in efficiency. At a point, the increased strain outweighs the benefits of the larger active area, making the rigid fixation more effective at maintaining volume displacement. This explains why the two data lines intersect as the withdrawn volume grows larger.

In its current form, Configuration 1 displaces a larger volume than Configuration 2. Configuration 1 achieves a maximum volume displacement of 16.48 ± 0.21 mL (SD, n = 5) with an input change volume of 7.6 mL, while Configuration 2 displaces 15.79 ± 0.36 mL at the same input volume, yielding a difference of 5.37%. This difference arises because the last turn of the device in Configuration 2 functions purely as a counter‐twist mechanism and does not contribute to aortic compression, reducing the effective length of the artificial muscle. However, this small volume difference translates to a small difference in arterial pressure, which is acceptable and can be mitigated by increasing the number of muscle turns. This difference is further attenuated by the plateauing effect observed in both configurations, where volume displacement levels off after a certain point of muscle contraction. A general trend observed across both configurations is that the initial relationship between withdrawn volume and volume displaced is linear. However, beyond a certain threshold, further decreases in input volume yield diminishing returns in stroke volume. This is because the aorta's cavity becomes fully compressed, leaving no more fluid to displace. At this point, further shortening of the hydraulic artificial muscle causes the muscle to press directly against the aorta, potentially causing harm to the aortic tissue. Given this information, the plateauing effect also defines the device's optimal working range, within which operation must be maintained to prevent tissue damage. For Configuration 1, the plateau occurs at a change in input volume of ≈4.5 mL, corresponding to an output stroke volume of 13.69 mL. In Configuration 2, the plateau occurs at ≈4.9 mL of change in input volume, yielding a stroke volume of 11.41 mL. These values indicate the points at which both configurations reach their maximum effective volume displacement and operation beyond these thresholds should be avoided for safety considerations.

Interestingly, in Figure 5c, we observe a special case where the performance of the flexible fixation is comparable to, or even exceeds, that of the rigid fixation at larger muscle contractions. In previous analyses and comparisons, the volume displacement of the device was evaluated without considering the twist in Configuration 1. All the comparisons primarily focused on the no‐twist performance of prototype 1 and prototype 2. However, as introduced above, Configuration 1 can induce a twist in the underlying aorta. The maximum stroke volume achieved by Configuration 1 with the twist reached 16.86 mL, compared to the benchmark's 16.45 mL. This highlights the advantage of incorporating twisting into the device, as the twist engages more sections of the aorta beyond what is enclosed within the device, increasing the active area and resulting in improved volume displacement. However, this large angle of twist is not suitable for cardiac applications, as it forces the artery to behave in an unnatural way, which will almost certainly lead to inflammation and fibrosis after longer‐term implantation. While beneficial in terms of performance, this effect is better suited for applications involving biological tissues that can tolerate more deformation, such as the colon, where structural flexibility and movement are less of a concern compared to sensitive tissues like arteries.

Building on these findings, we further tested the device's volume displacement performance at pressures ranging from 20 to 140 mmHg using Configuration 2: the counter‐twist design, which operates at its maximum defined range. These experimental pressures cover hypotensive to hypertensive aortic pressure, simulating reduced, physiological, and elevated conditions. As shown in Figure 5d, stroke volume decreases as pressure increases, dropping from 13.40 mL at 20 mmHg to 11.36 mL at 140 mmHg. This 2.04 mL reduction occurs because the device must overcome greater resistance at higher pressures, where the aorta presents a larger force that opposes compression. Despite this decrease in stroke volume, the device consistently displaces significant volume across the pressure range, demonstrating its ability to operate both effectively and reliably. This is reinforced by the small standard deviations observed in each trial. Since individual variations in aortic pressure fall within the tested range, the results suggest that the EACD can reliably deliver counterpulsation across different physiological conditions. Counterpulsation therapies are effective primarily in hypotensive scenarios. In cases of elevated blood pressure, further increases could lead to adverse effects. Testing the device across a wide range of pressures, including those beyond the target hypotensive range, aims to provide valuable insights into stroke volume trends and inform potential future improvements. Stroke volume characterization was performed at 120 mmHg as a benchmark, which exceeds the typical peak systolic pressure in hypotensive scenarios (≈ 90 mmHg). As a result, the device may exhibit greater stroke volume under clinically relevant hypotensive conditions.

Finally, when compared to developing counterpulsation solutions, the device in this study demonstrates a strong stroke volume performance at high pressures, as shown in Table S5 (Supporting Information). However, when compared to more established devices like the IABP, its stroke volume becomes a limitation. The IABP, which is considered one of the most effective counterpulsation devices, achieves stroke volumes ranging from 30 to 50 mL, largely because it is deployed in the descending aorta, where there is more space for volume displacement. In contrast, the device in this study targets the ascending aorta, which is limited by its shorter length. Another ascending aorta‐targeting device, the C‐Pulse, displaces 20 to 30 mL of blood, which is made possible by its design that maximizes the active area in contact with the aorta. This suggests that with design optimizations, such as increasing the contact area or modifying the shape, the device presented in this study could achieve a more competitive stroke volume.

### 2.4 In‐Vitro Evaluation of Hemodynamic Improvements Induced by the EACD

#### 2.4.1 Experimental Setup and Rationale

**Figure 6:**
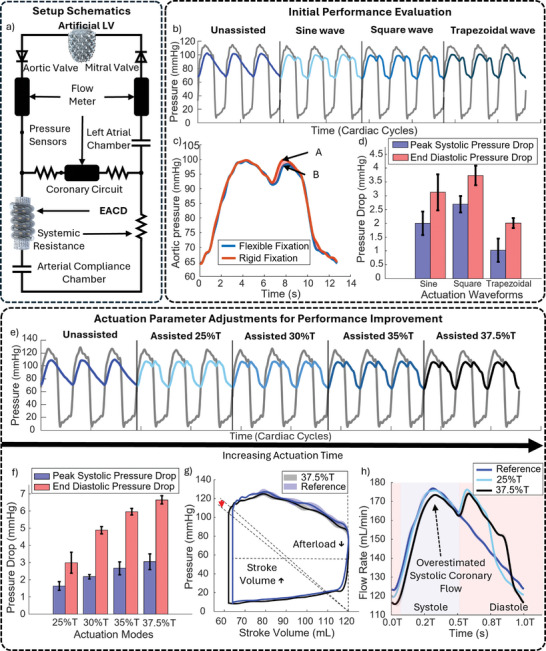
In Vitro Evaluation of Device Performance a) Schematic of the mock circulation loop used for testing.b) Pressure waveforms for unassisted and assisted conditions under various input actuation waveforms. c) Comparison of pressure waveform between flexible and rigid fixation across an averaged cardiac cycle. d) Pressure drop measurements across different actuation input waveforms. e) Pressure waveforms for unassisted and assisted conditions under increasing actuation durations, T is the time for one complete cardiac cycle. f) Pressure drop measurements for different actuation durations. g) Pressure–volume loop illustrating afterload reduction and stroke volume increase. h) Variations in simulated coronary flow rate over the cardiac cycle under constant coronary resistance conditions, induced by unassisted (ref) and assisted (20%T and 35%T) pressure waves.

To further evaluate the device's efficacy in providing hemodynamic stabilization in vitro testing was conducted using a MCL. This MCL was designed to simulate the left‐side circulation of the cardiovascular system under conditions that closely resemble those encountered in these critical care scenarios. **Figure** **6**a provides a simplified illustration of the test system setup, with its corresponding CAD model available in Figure S5 (Supporting Information). The MCL mimics blood flow from the left ventricle to the left atrium, creating a controlled environment that accurately replicates human physiological conditions.

One of the key features in this MCL setup is the biomimetic artificial left ventricle(ALV), which replicates natural contractile motion and recreates physiological hemodynamic conditions.^[^ ^39^ ^]^ This model enables simulations of both healthy and pathological cardiac states, providing an adaptable platform for evaluating the device's therapeutic effects under a range of clinical scenarios (Figure 6a). To simulate compromised cardiac conditions, the ALV is specifically tuned to exhibit reduced contractility, resulting in a decreased stroke volume that mirrors the target clinical scenarios. Incorporating this robotic left ventricle into the MCL setup introduces a dynamic, interactive testing environment. The artificial ventricle is capable of responding to hemodynamic changes in the ascending aorta, establishing a feedback loop that approximates natural physiological responses. This level of fidelity supports a more accurate assessment of the EACD's potential to stabilize hemodynamics under realistic cardiac conditions.

Additionally, an artificial aorta segment, engineered to match the mechanical and geometric properties of the human aorta,^[^ ^40^, ^41^ ^]^ was incorporated into the MCL to provide a realistic platform for the EACD's counterpulsation, enabling natural interactions with the aortic wall. A coronary circuit was also added to evaluate the device's impact on coronary perfusion, a crucial factor for stabilizing hemodynamics in the target patient populations. Together, these components work with the artificial left ventricle to form a comprehensive physiological model for assessing the EACD's effectiveness. This system captures pressure changes and flow dynamics, enabling a quantitative assessment of the device's effects on the cardiovascular system.

To simulate a hypotensive environment characteristic of cardiogenic shock patients, the aortic pressure was tuned to ≈ 95 mmHg with a diastolic pressure of ≈ 65 mmHg by adjusting the systemic resistance and arterial compliance of the MCL. These pressures correspond to reduced pulse pressure, negatively affecting tissue perfusion. As a counterpulsation device, the device stabilizes hemodynamics by counteracting reduced pulse pressure by enhancing coronary blood flow and reducing afterload. By improving myocardial oxygen and nutrient delivery while decreasing ventricular workload, the device aims to increase stroke volume and restore aortic pressure to physiological levels.^[^ ^42^ ^]^ These parameters were closely monitored throughout the in vitro tests to assess the device's therapeutic potential.

Testing was conducted at ≈4.5 beats per minute (bpm) due to limitations in the MCL. Specifically, while all water tubes in the MCL have a diameter of 25 mm, the flow meter used in the setup has a bore diameter of only 12.7 mm. These water tubes act as conduits that support systemic circuit flow, simulating the pathways through which blood circulates in a physiological system. Furthermore, although the ALV demonstrates competency in reproducing many physiological responses, it falls short in operating at high speeds due to design limitations and the components available. The ALV employs check valves with an inner diameter of 6 mm to replicate the functions of the mitral and tricuspid valves. Ejections from the ventricle must pass through this narrow component before entering the systemic circulation circuit. These reductions in diameter increase flow resistance and create a local pressure buildup in the circuit, with the effect accentuating at higher flow speeds. Such narrowing induces a stenotic effect, resembling the impact of physiological stenosis, where a narrowed vessel or valve restricts flow and elevates pressure, though it arises here mechanically rather than anatomically. As a result, the testing speed of MCL is tuned to 4.5 bpm max where some stenotic effects were observed but remained within a reasonable range(<20mmHg). Further increases in heart rate exacerbated these stenotic effects, causing severe pressure drops across the “stenotic” valves and flow meters, leading to unphysiological measurements.

Regarding the device itself, the muscles have been statically examined for their radial force production under high‐frequency conditions in modular tests. The muscles demonstrated the ability to produce consistent force at rates as high as 90 bpm (1.5 Hz), with previous studies showing they are capable of reaching up to 20 Hz.^[^ ^27^ ^]^ However, with increasing frequency, there is also an increase in hysteresis, which could affect the overall performance at higher operational speeds. Under dynamic conditions, such as when the underlying structure (e.g., the aorta) changes its radius during compression, the force production profile may be further influenced. With further improvements to the testing setup, higher‐speed validation will become feasible, and future work will focus on studying the dynamic behavior of the muscles at higher frequencies.

To validate the test system output at slower operational speeds, a computational model was employed to assess the test platform independently of the EACD. Figure S6c (Supporting Information) presents the lumped parameter mechanics model simulating the cardiac left ventricle coupled with the systemic circulation.^[^ ^43^ ^]^ The Pressure–Volume (PV) loops for heartbeats at 1 bpm and 60 bpm^[^ ^44^ ^]^ demonstrate alignment when scaled with parameters such as compliance and resistance. The simulated aortic pressure, derived by solving the model's differential equations at both heart rates, shows strong agreement in waveform and magnitude. This provides compelling evidence that the MCL system's results at slower speeds, such as 4.5 bpm, reliably represent the physiological responses expected at higher heart rates, such as 60 bpm, through appropriate parameter scaling.With this computational verification supporting the use of parameter scaling in the MCL, in vitro assessments of EACD performance were subsequently conducted.

#### 2.4.2 Initial Performance Evaluation

Conventionally, square waves are employed to control the actuation pattern of counterpulsation devices.^[^ ^7^, ^42^, ^45^ ^]^ Figure 6c illustrates the aortic pressure when the device is actuated with a square wave, where the actuation state occupies 25% of the entire cardiac cycle (T). This 25% actuation period serves as a preliminary starting point, recognizing that the optimal actuation period can vary significantly among individuals due to differences in their cardiac conditions, which affect the time required for augmented pressure to fully stabilize. The figure shows a rise in aortic pressure after the dicrotic notch, which is directly attributed to the volume displacement caused by the device. This pressure augmentation mirrors the response seen in other established counterpulsation devices, indicating that the device can successfully induce the desired pressure difference.

Figure 6c also highlights the differences in output between the device using flexible fixation and device using rigid fixation. Operating within the working range determined in the previous integration test, the device with flexible fixation produces a pressure rise that is 1.35 mmHg lower than the rigid fixation method (as shown at points A and B on the plot). This difference is attributed to a 3.29 mL reduction in the volume displaced per stroke by the device, representing a 10.94% decrease in the induced pressure rise. This underscores the critical role that stroke volume plays in counterpulsation therapy, as small reductions in volume displacement, can significantly impact therapeutic outcomes. These findings provide a clear direction for future work, focusing on optimizing stroke volume to enhance the augmentation effect of the device.

The device was also actuated using trapezoidal and sine waves to explore potential control optimizations. The input signals and their actuation timings are illustrated in Figure S7a (Supporting Information). This test aimed to identify which waveform provides the most effective counterpulsation, thereby improving overall cardiac function. The resultant aortic and ventricular pressures for each input waveform are displayed in Figure 6b. In the Figure, we observe the aortic pressure differences between the unassisted condition, sine wave, square wave, and trapezoidal wave respectively.

Figure 6d summarizes the drop in aortic peak systolic pressure (AOSP) and aortic end‐diastolic pressure (AOEDP) produced by different input waveforms. It is evident that the square wave outperforms the other waveforms, producing a diastolic pressure drop of 3.61 mmHg, which is 27.78% more than the sine wave and 42.78% more than the trapezoidal wave. The superior performance of the square wave arises from its ability to more precisely and rapidly adjust pressure through effective control of stroke volume. The timing of actuation plays a critical role, with the prompt response of the square wave yielding better output. In contrast, the trapezoidal wave's reduced effect, despite the device contracting by the same amount as the other waveforms, can be attributed to the gradual rise in induced pressure. The trapezoidal wave causes a slower buildup in pressure before reaching maximum stroke, which leads to a pressure rise that settles before influencing the subsequent cardiac cycle. This also explains why the sine wave is more effective than the trapezoidal wave; the sine wave allows the device to reach maximum stroke volume faster after mitral valve closure, creating a larger induced pressure rise.

Through this preliminary test, we can conclude that the device can competently reduce afterload, as reflected by the drop in AOPSP and AOEDP. The square wave remains the most effective actuation profile due to its ability to generate higher augmented pressure. However, the achieved aortic pressure is still too low to be sufficiently beneficial in a hemodynamically unstable situation. Therefore, further optimizations are required to increase the reduction in afterload and improve the device's therapeutic effectiveness in critical care scenarios.

#### 2.4.3 Parameter Adjustments and Response Analysis

An investigation was conducted to assess the effect of extended actuation duration on the device's performance to optimize its afterload reduction. Actuation timing is critical for the device's effectiveness. The onset of the actuation signal is synchronized with the dicrotic notch in the cardiac cycle, while the offset timing needs to be adjusted for patient‐specific conditions. In this experiment, various actuation durations were tested. The aortic and ventricular pressures during device assistance, actuated by square waves with differing durations, are shown in Figure 6e. Longer actuation periods delayed the settling of increased aortic pressure, leading to a more pronounced effect on the subsequent AOSP and AOEDP. These changes are summarized in Figure 6f, showing a clear trend: increasing the actuation time results in a more substantial reduction in afterload. The maximum drops in AOPSP and AOEDP, 3.32 mmHg and 6.32 mmHg respectively, were achieved with a 37.5% actuation duration. The extended actuation sustains aortic pressure, and when released just before systole, it creates a vacuum that reduces the afterload the heart must overcome in the next contraction, improving cardiac efficiency and lowering the workload. However, these values may not fully reflect the device's actual afterload reduction due to stenosis in the test setup, patient‐specific factors such as cardiovascular conditions and aortic properties can also affect outcomes. Instead, this test proves that adjusting actuation timing based on individual vascular compliance and resistance could significantly enhance therapeutic efficacy.

The device was also tested under delayed actuation conditions. The aortic pressure response mirrored the late actuation profile of other counterpulsation devices, such as the IABP,^[^ ^46^ ^]^ as shown in Figure S7b (Supporting Information). In this case, afterload increased, demonstrating the negative effects of improper actuation timing. This replication of the late actuation pattern reinforces the device's counterpulsation capabilities from another dimension and highlights the importance of precise timing for maximizing therapeutic outcomes.

To demonstrate the device's impact on the ventricle, a pressure–volume (PV) loop is shown in Figure 6g, comparing assisted and unassisted cardiac cycles. In clinical practice, PV loops are commonly used to assess ventricular function. Figure 6g shows an increase in stroke volume, indicated by the horizontal dotted line, and a reduction in afterload, reflected by the decreased slope of the other dotted line after EACD use. This finding reveals a trend consistent with studies on the effects of counterpulsation therapy on ventricular function, as noted by Klabunde et al.,^[^ ^47^ ^]^ supporting the device's therapeutic potential.

The EACD was tested under conditions of mild stenosis, with a peak pressure gradient of 20 mmHg between the ventricle and aorta. Stenosis reduces the effectiveness of afterload reduction by increasing aortic resistance, which forces the heart to work harder and limits the full benefits of counterpulsation therapy.^[^ ^48^ ^]^ As a result, the EACD's impact on afterload reduction may not be fully transferred to the ventricle and could be underrepresented in this study. In a non‐stenotic scenario, a more pronounced effect would likely be observed, as indicated by the greater AOEDP drop with a 25% actuation duration in a less stenotic system shown in Figure S7c (Supporting Information).

Additionally, the artificial left ventricle (ALV) used in this experiment is less responsive to afterload changes than a natural ventricle, particularly with small adjustments. Only substantial changes in afterload elicit a marked response in the ALV, which follows an exponential response pattern.^[^ ^39^ ^]^ This is due to the mechanical properties of the soft artificial muscles do not fully replicate the physiological behavior of myocardial muscle fibers. Therefore, the PV loop in this study serves as an indication of the device's potential therapeutic effect. It sufficiently demonstrates the device's capability, though it should be interpreted as showing a trend rather than providing precise quantitative data.

Finally, we investigated the increase in coronary flow induced by the device. While advanced MCLs use dynamic coronary resistance mechanisms to simulate realistic conditions,^[^ ^49^, ^50^ ^]^ this experiment employed a constant resistance model to focus on broader hemodynamic trends. The resulting coronary flow, shown in Figure 6h, represents coronary flow under three conditions: unassisted, assisted with 25% actuation duration, and assisted with 37.5% actuation duration. The flow waveform reflects the pressure gradient between the coronary and return circuits as it drives the flow.

Under physiological conditions, most coronary flow occurs during diastole due to systolic ventricular contraction increasing resistance in the coronary arteries. However, with the simplified constant coronary resistance, systolic flow is overestimated. Meanwhile, the diastolic flow profile which is the region of interest where the EACD can have an effect remains valid. The maximum coronary flow is tuned to match the literature flow rate^[^ ^51^ ^]^ to approximate the coronary flow profile. Note that transient data between systolic and diastolic flow is also affected by the systolic flow overestimation.

Although the setup's limitations prevent precise quantification, the diastolic flow waveforms indicate that extending the device's actuation duration increases coronary blood flow. The observed enhancement in coronary flow is driven by the increased pressure gradient during diastole compared to the unassisted case, as illustrated in Figure S8a (Supporting Information). Moreover, Figure S8b (Supporting Information) shows the normalized increase in coronary flow at different actuation durations, providing further evidence of this relationship. While this simplified setup does not fully capture coronary dynamics, this experiment validates the claim that the device can augment the coronary flow pattern to promote cardiac stabilization.

### 2.5 Discussion

#### 2.5.1 Comparison with Existing Devices

The in vitro and in vivo performance of existing technologies, along with their limitations and unique advantages, are summarized in Table S1 (Supporting Information). While diastolic pressure change would ideally serve as a comparison metric to directly reflect the device's counterpulsation efficacy, it is highly sensitive to variations in test conditions. Parameters of the test system, such as pulse pressure, can significantly impact the response to the same device, leading to inconsistencies. Therefore, stroke volume becomes a more standardized comparison metric for evaluating different devices, as pressure changes are largely determined by each device's volume displacement capability. Under physiological pressures (90 to 120 mmHg), our device achieves the highest stroke volume among non‐pneumatic actuation technologies. Many existing systems, such as those utilizing magnetic or electrical forces, struggle to provide sufficient volume displacement for meaningful pressure impact. However, compared to established devices like the intra‐aortic balloon pump (IABP), Kantrowitz CardioVAD (KVC), and C‐Pulse, our device's stroke volume remains lower.

IABPs achieve stroke volumes of 30–50 mL,^[^ ^52^, ^53^, ^54^ ^]^ facilitated by their placement in the descending aorta via a percutaneous approach,^[^ ^7^ ^]^ while C‐Pulse, targeting the ascending aorta, reaches only 20–30 mL.^[^ ^14^ ^]^ The Kantrowitz CardioVAD (KVC) achieved the largest stroke volume of 60 mL^[^ ^54^ ^]^ due to its descending aorta placement. The spatial limitations of the ascending aorta constrain stroke volume, leading to a weaker vacuum effect and reduced afterload reduction, which limits the efficacy of devices like ours and the C‐Pulse compared to IABPs. Newer extra‐aortic devices aim to avoid direct blood contact to reduce the risk of infections and thromboembolic events while providing support from days to weeks rather than just hours.^[^ ^55^, ^56^ ^]^ This necessitates external placement to the aorta, and accessing the descending aorta requires more invasive procedures. This presents a trade‐off: while the descending aorta allows for larger stroke volumes, the procedures are highly invasive. Devices targeting the ascending aorta, like ours, sacrifice stroke volume for a less invasive approach.

#### 2.5.2 Device Placement: Proximal Versus Distal

The influence of device placement along the aorta is critical. Numerous studies have demonstrated that positioning augmentation devices in the ascending aorta (proximal placement) enhances the augmentation effect compared to placement in the descending aorta (distal placement).^[^ ^57^, ^58^, ^59^ ^]^ This enhancement is attributed to the more immediate transfer of pressure, reducing dampening from wave reflections^[^ ^60^ ^]^ and minimizing latency in pressure response near the aortic valve, allowing for precise diastolic support synchronized with the cardiac cycle.^[^ ^61^ ^]^

Proximal placement also reduces the risk of vascular trauma by avoiding interference with major branch vessels, such as the renal and mesenteric arteries, thereby lowering the likelihood of complications like ischemia. While proximal placement may limit stroke volume due to reduced augmentation space, it compensates by directly enhancing coronary perfusion and diastolic pressure. This strategy, adopted by devices like C‐Pulse, prioritizes coronary perfusion over the potentially greater ventricular unloading achievable with distal placement. Given the EACD's intended use in cardiogenic shock with myocardial infarction, its ability to improve coronary perfusion and stabilize myocardial oxygenation is critical.

In contrast, distally placed counterpulsation devices may achieve larger stroke volumes but at the expense of attenuation during pressure transfer to the ventricle and coronary circuit. These findings highlight that stroke volume alone does not determine augmentation efficacy; placement and pressure dynamics are equally critical. Future in vivo studies will incorporate these factors to fully evaluate the EACD's functionality and optimize its design.

#### 2.5.3 Actuation Medium and Operational Frequency

Another critical factor influencing device performance is its ability to operate effectively at clinically relevant frequencies. Pneumatically actuated devices, such as the C‐Pulse and intra‐aortic balloon pump (IABP), respond quickly to high‐frequency signals. However, their fast response times often come at the cost of significant energy loss and reduced precision due to air compressibility, leading to signal attenuation at higher frequencies. Other actuation methods, such as electric and magnetic systems, have also been explored.^[^ ^19^, ^62^ ^]^ Electric actuators, for instance, can operate at frequencies of several kHz, but their limited range of motion and energy loss to heat generation pose challenges, particularly in biological environments. Similarly, while magnetic actuation shows theoretical promise, its force output at clinically feasible distances is often insufficient for practical applications.

In comparison, the hydraulic system used in our EACD operates more slowly than other devices using pneumatic systems. This is due to the limitation of our mechanical driving source rather than the EACD itself. Compared to pneumatics, hydraulics offer precise and stable control with a linear input‐output relationship, enabling predictable and safe actuation. This quality makes hydraulic systems advantageous in medical applications that prioritize control over speed. In addition, hydraulic systems are well‐suited to cardiac frequencies, providing reliable operation with reduced noise and bulk. The EACD demonstrates these advantages, requiring only 7.6 mL for actuation, offering the device miniturization and potential for full implantability. At its current stage, the EACD faces limitations in achieving high‐frequency actuation due to its larger hysteresis. However, with further design optimizations, this constraint could be mitigated to enhance performance, presenting a promising area for future exploration.

#### 2.5.4 Clinical Considerations

While the primary scope of this study is to investigate the working principles and hemodynamic augmentation capabilities of the EACD, its proposed clinical application introduces inherent challenges, including tissue remodeling, inflammation, and safe implantation. Addressing these concerns fully will require validation through in vivo studies. However, preliminary insights from the literature provide an expected framework for understanding the potential outcomes.

Tissue remodeling is unlikely to occur with the EACD. Remodeling typically results from sustained deviations from baseline conditions, such as chronic mechanical loading or inflammation,^[^ ^63^ ^]^ neither of which are inherent to the EACD's operation. By design, the EACD allows the aorta to return to its natural state after each cycle of external compression, minimizing sustained mechanical stress or tissue changes. Supporting evidence comes from chronic in vivo studies of the C‐Pulse device, which demonstrated minimal tissue remodeling over a 12‐month period. Remodeling observed in those cases stabilized within the first month and showed no further progression.^[^ ^64^ ^]^ Furthermore, the EACD's intended short‐term use, typically limited to days or weeks, further reduces the likelihood of significant remodeling. Clinical studies have also shown that remodeling is reversible upon device removal.^[^ ^65^ ^]^ These findings suggest that the extent of remodeling associated with the EACD is expected to be minimal and not sustained long‐term.

Inflammation is another critical concern when placing a device around the aorta, given the tissue's sensitivity. Adverse inflammatory responses can lead to severe complications, such as pericardial effusions or pericarditis. However, the EACD's design minimizes these risks. The device's most proximal portion terminates above the sinotubular junction, avoiding the pericardial reflection and significantly reducing the likelihood of direct interference with the pericardium. In future work, we also plan to integrate a silk‐based biomaterial currently under development as a biological interface. This interface is expected to mitigate sensitivity and reduce inflammation.

Patient selection will play a pivotal role in minimizing risks associated with the EACD. The cyclic compression exerted by the device poses potential hazards for individuals with fragile or diseased aortic tissue, where rupture could have fatal consequences. Contraindications adapted from clinical studies of the C‐Pulse device include conditions such as ascending aortic calcification, previous aortocoronary bypass grafts, a history of aortic dissection, connective tissue disorders like Marfan's syndrome, and prior interventions such as repaired aortic coarctation, composite grafts, or aortic root replacement.^[^ ^66^ ^]^ Additional exclusions include moderate to severe aortic insufficiency, prior LVAD implantation, heart transplantation, and significant carotid artery stenosis. These guidelines provide a valuable framework for patient selection during future clinical development.

Furthermore, the implantation procedure for the EACD demands meticulous surgical planning and evaluation. Although a preliminary implantation guide is included in the supporting information, it primarily addresses the procedural aspects of device deployment, with limited focus on surgical considerations. Implementing the device in a living being presents significant surgical challenges, including determining the appropriate incision size, safely separating the aorta from surrounding tissues, and selecting an optimal incision location to minimize trauma and improve surgical access. Each of these factors plays a critical role in ensuring the safety and success of the procedure. A key focus of future clinical studies will be the exploration of minimally invasive implantation techniques, which align with the EACD's design objectives of minimizing recovery time and procedural risks.

#### 2.5.5 Durability

Durability is a critical factor for medium‐term devices and a preliminary testing over 10 000 cycles under dynamic conditions has provided initial insights into the current design's performance (Section S10, Supporting Information). While no failures were observed during the tested cycles, a gradual decline in performance highlights areas requiring refinement.

Key improvement strategies include substituting materials in the artificial muscle to reduce wear, optimizing bonding techniques for better integration with POF and the stabilization mechanism, and refining the design to minimize mechanical stress under cyclic loading. These adjustments, guided by experimental findings, aim to enhance durability and address the current prototype's limitations and will be further explored in future studies. However, challenges such as fatigue and wear are inherent to dynamic systems and cannot be entirely eliminated.

## 3 Future Directions and Conclusion

This feasibility study of the proof‐of‐concept device demonstrates the potential for a minimally invasive, non‐blood‐contacting counterpulsation solution. Designed to provide medium‐term hemodynamic support, the device targets patients with cardiogenic shock, acute decompensated heart failure, and post‐cardiac surgery recovery. Initial modular and in vitro tests show promising results in achieving automated deployment and hemodynamic augmentation at physiological pressures, supporting the need for further investigation.

While encouraging, this study identifies key challenges that require further refinement, including optimizing operational speed, enhancing stroke volume, and improving device durability. Future in vivo studies will be critical in addressing important clinical considerations, such as evaluating the device's long‐term effects on aortic tissue, potential inflammatory responses, and refining surgical protocols to ensure safe and effective implantation. These investigations will provide essential data to guide design improvements and support the device's progression toward clinical application.

With continued development and validation, the EACD could offer a viable, medium‐term extra‐aortic counterpulsation therapy. Its advancement holds promise for improving the management of hemodynamically unstable conditions and enhancing patient outcomes.

## 4 Experimental Section

The experimental setup for the documented experiments are presented in Figures S10 and S5 (Supporting Information).

### TGS Growth Pressure and Geometrical Accuracy Measurement

A pressure regulator (Supercheap Auto, AU) was used to control the pressure within the TGS, monitored by a pressure sensor (Honeywell, USA). The geometrical path of the TGS was tracked using a magnetic tracking system (3D Guidance TrakSTAR, NDI, EU) which provided x, y, and z coordinates. Pressure and 3D position data were collected and analyzed using MATLAB (MathWorks, USA).

Growth pressure measurements were taken using a TGS with a diameter of 13 mm, and a length of 75 mm, and configured helically. Initially, the TGS was fully everted, and as the pressure regulator knob was turned, the pressure within the TGS body increased, and this pressure was recorded. TGS growth was indicated by a rapid drop in pressure, easily identifiable on the pressure versus time plot. Subsequently, the same TGS was used to measure the impact of adding a stabilization mechanism. The TGS was everted again for growth, and each test was repeated three times to determine standard deviations.

For measuring the geometrical accuracy of the TGS, the pressure was set just above the growth pressure determined in the previous test (≈ 35 *kPa*). The base of the TGS was fixed to the table to prevent drifting during growth.

### Attachment Mechanism Strength Test

Two stabilization mechanisms were tested: Velcro (Velcro, UK) and Dual Lock (Alfa Lock, Velcro, UK). Each mechanism was trimmed to a size of 10 mm × 10 mm. Load cells (FUTEK, USA) measured the shear force each stabilization mechanism could withstand before disengaging. The test patches were made from thin polyvinyl chloride (PVC) boards trimmed to 20 mm × 40 mm strips, with a layer of POF taped to the surface to resemble the attachment between the TGS and the stabilization mechanism in the device. A high‐power linear slider (Zaber, Canada) produced precisely controlled displacement to apply the required stress to the stabilization mechanisms.

In the max shear force test, the load cell was attached to the 3D‐printed structure and fixed to the optical table (McMaster– Carr, US) by screws. One PVC strip was joined to the load cell, and its paired strip was screwed to the linear actuator. The linear actuator platform receded to increase the shear force acting on the junction point until failure. The same experimental setup was used for the durability test, except the linear slider input was substituted with sinusoidal waves over 2000 cycles of small amplitude. The starting shear force for the durability test was 70% of the maximum shear force, corresponding to a linear slider displacement of 6 mm.

### Contractile Unit Characterization

To measure the radial force a single hydraulic artificial muscle can produce in a helical configuration, four turns of the muscle were wound around a 3D‐printed cylindrical structure housing the load cell (FUTEK, USA). The cylinder diameter was 29 mm, corresponding to the physiological dimension of the ascending aorta.^[^ ^25^ ^]^ A linear slider (Zaber, Canada) controlled the input volume into the muscle by pushing the plunger of a 1 mL syringe (BD, USA). A pressure sensor (Honeywell, USA) monitored pressure changes as the input volume varied.

The radial force test was conducted using an input signal with a 7.5 mm amplitude and a period of 5s. The same experimental setup was used for the frequency test of the muscle, with the working range determined from the previous benchmark radial test. The amplitude of the signal was reduced to 4.5 mm. The test was performed at frequencies of 30, 45, 60, 75, and 90 bpm.

### Device Integration Test

The tests were performed using the experimental setup shown in Figure 5. An artificial aorta was fabricated to replicate the geometry and mechanical properties of the ascending human aorta, with water height used to simulate aortic pressure. The model was constructed using cotton fibre‐integrated Dragon Skin 30 silicone (Smooth‐On Inc., PA), chosen for its mechanical properties closely matching those of the human aorta, as documented in the literature.^[^ ^40^, ^41^ ^]^ Cotton fibres (Bunnings, AU) were added to simulate the aorta's extracellular matrix,^[^ ^67^ ^]^ allowing controlled radial expansion during the cardiac cycle and preventing rupture or tearing. The resulting aorta phantom had a 2.5 mm wall thickness and a 29 mm outer diameter.

The fabrication process started with the design of a 3D‐printed mold, consisting of an inner cylinder and two outer pieces. When assembled, this mold created a cavity for casting the aorta phantom. A thin layer of Dragon Skin 30 was first poured over the central mold, which had a fabric nest stretched over it to integrate the fibres into the phantom. Once the fabric was embedded, the mold was assembled and filled with silicone to achieve the final structure, ensuring the desired thickness and mechanical properties.

The device stroke volume characterization was performed under a constant pressure of 120 mmHg. Flow was measured using a flow meter (Atrato, RS, USA), and a pressure sensor (Honeywell, USA) was located just above the artificial aorta to monitor the working pressure. The stroke volume test involved pressurizing the artificial muscles to their most elongated form, then withdrawing volumes to compress the underlying aorta. The volume change corresponds to a displacement of 2 mm, repeated at every 2 mm increment up to 22 mm (7.6mL). The same procedure was performed for the test of device configuration 2.

Furthermore, the device was tested for its stroke volume at increasing aortic pressure using device configuration 1, without a twist. By changing the height of water in the column, different pressures were produced for the device to contract against. The corresponding water height levels were 272, 544, 816, 1088, 1360, 1632, and 1904 mm, respectively.

### In Vitro Test

This experiment utilized a MCL to replicate the flow dynamics and pressure conditions of the human cardiovascular system. The setup was based on an artificial left ventricle test model, with an additional circuit for coronary flow. Coronary resistance was simplified to a constant resistance value by partially blocking the tube using a Hoffman clamp (AliExpress, CN), adjusted to achieve a maximum coronary flow of around 150 mL/min, corresponding to physiological flow values.^[^ ^51^ ^]^ Flow into and out of the ventricle, as well as coronary flow into the return circuit, was measured using a flow meter (Atrato, RS, USA). These measurements allowed for the computation of the work done by the ventricle and the effect of the device. Pressures in the ventricle, coronary circuit, and aorta were monitored to ensure accurate physiological flow conditions and study the device's effect. Parameters such as aortic pressure, ventricular pressure, and pulse pressure were tuned by adjusting systemic resistance and arterial compliance. The target working range for aortic pressure was set between 95–65 mmHg to mimic the cardiac condition of HF patients.

The first in vitro experiment investigated the device's effect using different input waveforms. The actuation time for each signal was identical, with all signals occupying 25% of the cardiac cycle. The aortic pressure measurements indicated the pressure change induced by the device, and the coronary artery flow measurements showed an increase in flow due to the device. For the optimization test, the device was tested with increasingly longer actuation duration of 25%, 30%, 35%, and 37.5% of the cardiac cycle. The same sets of data are collected, including the ventricular and aortic pressure, and volume flowing through the coronary, aortic and venous return branches.

## Conflict of Interest

The authors declare no conflict of interest.

## Supporting information

Supporting Information

Supplemental Video 1

Supplemental Video 2

Supplemental Video 3

## Data Availability

The data that support the findings of this study are available from the corresponding author upon reasonable request.
