# Supplementary material for: Development of a Self‐Deploying Extra‐Aortic Compression Device for Medium‐Term Hemodynamic Stabilization: A Feasibility Study
Source: Adv Sci (Weinh). 2024 Dec 27;12(11):2412120. doi: 10.1002/advs.202412120 (PMC11923917; doi:10.1002/advs.202412120)
Supplement: Supplementary file 1 — Supporting Information [file ADVS-12-2412120-s002.pdf]

ADVANCED SCIENCE
Open Access

Supporting Information

for *Adv. Sci.*, DOI 10.1002/advs.202412120

Development of a Self-Deploying Extra-Aortic Compression Device for Medium-Term Hemodynamic Stabilization: A Feasibility Study

*Adrienne Ji, James Davies, Phuoc Thien Phan, Chi Cong Nguyen, Bibhu Sharma, Kefan Zhu, Emanuele Nicotra, Jingjing Wan, Hoang-Phuong Phan, Christopher Hayward, Nigel H. Lovell and Thanh Nho Do**

Supporting Information

Development of a Self-Deploying Extra-Aortic Compression Device for Medium-Term Hemodynamic Stabilization: A Feasibility Study

*Adrienne Ji, James Davies, Phuoc Thien Phan, Chi Cong Nguyen, Bibhu Sharma, Kefan Zhu, Emanuele Nicotra, Jingjing Wan, Hoang-Phuong Phan, Christopher Hayward, Nigel H. Lovell, Thanh Nho Do**

A. Ji, J. Davies, Dr. P. T. Phan, C. C. Nguyen, B. Sharma, K. Zhu, E. Nicotra, J. Wan, Prof. N. H. Lovell, Dr. T. N. Do
Graduate School of Biomedical Engineering, Faculty of Engineering, and Tyree Institute of Health Engineering (IHealthE), UNSW Sydney, Kensington Campus, Sydney, NSW 2052, Australia

Dr. H. P. Phan
School of Mechanical and Manufacturing Engineering, Faculty of Engineering, UNSW Sydney, Kensington Campus, Sydney, NSW 2052, Australia

Dr. C. Hayward
Department of Cardiology, St Vincent's Hospital, Sydney, NSW 2010, Australia; St Vincent's Clinical School, Faculty of Medicine, UNSW, Sydney, NSW 2052, Australia

Corresponding Author: Dr. T. N. Do
Email Address: tn.do@unsw.edu.au

S1 Comparison with Existing Innovative Solutions

Table S1: Comparison with Existing Solutions.

Device	Working Principle	Augmentation Effect	Advantages	Limitations
IABP[1] [2] [3]	A minimally invasive intra-aortic pneumatic pump implanted through a catheterization procedure via the femoral artery. Typically displacing 30-50 mL of blood per inflation, depending on the patient's size.	In an in vivo trial targeting the treatment of cardiogenic shock caused by MI, a pulse pressure of 43 mmHg produced a 4 mmHg drop in AOPSP and an 8 mmHg drop in AOEDP.	1. Pneumatic actuators support high-speed actuation.<br>2. Lower risk profile than any other assistive devices.<br>3. Minimally invasive implantation.<br>4. Large stroke volume.	1. Low morbidity, requires intensive monitoring.<br>2. Low hemodynamic efficacy compared to VADs.<br>3. Provides highly limited duration of support.<br>4. Potential risk of complications (hemolysis, thrombosis infections etc.)
Kantrowitz CardioVAD [4]	A permanent version of IABP formed of a single layer of textured polyurethane with 60mL volume displacement sutured to the descending aorta.	The in vivo study targeting congestive heart failure with a pulse pressure of 45 mmHg, showed a 10mmHg rise in the augmented systolic pressure followed by a 5mmHg drop in AOEDP.	1. Provides long-term hemodynamic supports.<br>2. Largest stroke volume per inflation.<br>3. Internal blood pump reduces the risk of infection.<br>4. Coating and surface modifications for enhanced biocompatibility	1. Highly invasive implantation procedure.<br>2. Low hemodynamic efficacy compared to VADs.<br>3. Potential negative impact on the aorta's structure<br>4. The stabilization using suture makes removal of device challenging

WILEY-VCH

Device	Working Principle	Augmentation Effect	Advantages	Limitations
C-Pulse[5],[6]	A polyurethane balloon wrapping around the aorta inflate/deflate pneumatically driven by external pumps.	Displace volume of 20-30 mL at physiological pressure. The in vivo study conducted with 7mL SV caused a 2mmHg drop in AODBP, < 5mmHg drop in AOSBP, 67% increase in diastolic coronary blood flow.[7]	1. Pneumatic actuators support high-speed actuation.<br>2. Effective non-blood-contacting design.<br>3. Less invasive than ventricular assistive devices.<br>4. Large stroke volume	1. Require stringent patient screening.<br>2. Driveline infection.<br>3. Potential damage to the aorta after long-term compression.<br>4. Large operation noise.<br>5. Limited study on hemodynamic augmentation efficacy.
E-VaC[8]	Electrohydraulic (HASEL) sleeve driven by high voltage to augment the pulmonary artery in the case of right ventricular failure.	A drop of 8mmHg in peak systolic pressure at mPAP < 20mmHg (60 bpm). Diastolic pulmonary flow increased by 66.2%.	1. Lightweight and obviate drivelines.<br>2. Despite the use of high voltage, the current is constricted within the safety limit.<br>3. Failure mode does not impact the normal functioning of the artery.<br>4. High-frequency response	1. In vitro experiments were done without a contracting ventricle.<br>2. Limited ability to displace volume.<br>3. Low working pressure range.<br>4. Implantation methods have not been addressed.
Ferromagnetic Assist Device[9]	Micromagnetic particle-loaded silicone cuff actuated externally using magnets.	The device displaces 39ml at full contraction. 50% contraction against 50mmHg at 1.2T.	1. Lightweight and obviate drivelines.<br>2. Large stroke volume if can contract fully.	1. Lacking in vitro validation.<br>2. No study on the dynamic performance of the device.<br>3. Limited contraction at physiological aortic pressure.<br>4. Require strong magnetic field.<br>5. Uncontrolled contraction.

Device	Working Principle	Augmentation Effect	Advantages	Limitations
DEA Aorta[10]	A dielectric-elastomer aorta that replaces the ascending aorta with its stiffness controlled by voltage.	5 mmHg drop in AOEDP and 7 mmHg drop in AOPSP at peak systolic pressure of 105 mmHg with no change in average flow rate per cardiac cycle.	1. The electric field (12 kV) is fully confined within the device, minimizing the risk of tissue damage.<br>2. Potential for a fully implantable device powered by a Li-ion battery, expected to last up to 40 hours.<br>3. High response rate to high-frequency input signals.<br>4. Can serve as an aortic graft even if the device fails.	1. Limited operational lifetime of the DEA.<br>2. Variability in fabrication can lead to significant performance differences.<br>3. Safe operation is restricted to a pressure range between 65 to 105 mmHg.<br>4. Requires highly invasive implantation procedures.<br>5. Blood-contacting
The device proposed in the current paper	Helically arranged artificial muscles wrapping around the ascending aorta secured in place by a flexible vine and stabilization mechanism to induce periodic volume displacement.	Maximum stroke volume of 16.69 under 120 mmHg. 6.32 mmHg drop in AOEDP and 3.27 mmHg drop in AOPSP at peak systolic pressure of 110mmHg. Increase in coronary flow	1. Relatively large stroke volume<br>2. Minimally invasive implantation.<br>3. Automated device deployment.<br>4. Wide operation pressure range.<br>5. Samll muscle input volume provides the potential for full implantability.	1. Lack of validation on high-frequency dynamic device response.<br>2. Insufficient studies on the device's effects on biological tissues.<br>3. Require patient screening.

S2 Device Fabrication

The fabrication of the device utilizes the materials listed in Table S2. A modular approach is employed in the device's construction to facilitate rapid prototyping and rigorous preliminary testing, where failures are expected. This design allows for easy replacement and maintenance of individual parts during the development process.

Table S2: List of Materials Comprising Each Module of the Device

**Module**	**Component**	**Material & Specifications**
**Actuation & control**	Actuator driver	Zaber linear actuator (Zaber, Canada)
	Control algorithm	MATLAB & Simulink (MathWorks, USA)
**Deployment**	Tubular Growing Skin (TGS)	Polyolefin lay flat tube (AliExpress, China)<br>Material thickness: 6.35×10^-2 mm<br>OD before shrinkage: 30 mm<br>OD after shrinkage: 13 mm
	Deployment catheter	Clear Vinyl tubing (Bunnings, Australia)<br>OD: 16 mm<br>ID: 13 mm
	Pressurization chamber	Polycarbonate tube and sheets (Master-carr Supply Co., USA)<br>3D printing connector (Ultimaker, Netherland)<br>Smooth-sil 960 Gaskets (Smooth-on inc, USA)
	Feeder	SLA printed muscle feeders<br>PLA 3D printed gears<br>Pitch Diameter: 16.5mm<br>Number of teeth: 15
**Stabilization**	Adhesive	Heavy duty velcro (Velcro, Switzerland)<br>Force specification: 7kg per 50mm×100mm<br>Alfa lock (Velcro, Switzerland)<br>Force specification: 72 PSI
**Contraction**	HFAM	Coil Spring (McMaster-Carr Supply Co., USA)<br>OD: 3.18mm<br>ID: 2.51mm<br>k: 0.068N/mm<br>Rubber tube (Ebay, USA)<br>OD: 3.18 mm<br>ID: 1.59 mm<br>E(100%): 1.856MPa<br>5ml Syringe (Becton Dickinson, Australia)
	Medium transmission line	Fluid transmission tube (ColeParmer, USA)

Module	Component	Material & Specifications
		OD: 2mm
	Muscle end cap	PLA 3D printed cap (Ultimaker, Netherland)<br>Filament Diameter: 1.75mm<br>Cap OD: 13mm

S2.1 Actuation and Control

The device in its current form has not yet been tested for synchronization with cardiac signals. However, for effective operation as a cardiac assistive device, synchronization with these signals is crucial. At present, control of the device is achieved using a MATLAB script that communicates with the linear actuator through serial communication.
The system currently uses a feed-forward control approach based on the assumption of a regular and periodic heart rate, which implies a constant actuation period. This method does not account for natural variations in heart rhythm, which could limit the device's effectiveness in real-world scenarios where heart rates can be irregular. Future testing will explore synchronization with varying cardiac signals and the potential implementation of feedback control to adjust for these variations.
The specific triggering signals employed and the initial testing of the device under simulated conditions are discussed in detail in the in vitro investigation section. These preliminary tests provide insight into the device's response to controlled environments and highlight areas for further refinement.

S2.2 Deployment Module

The deployment module is a critical component of the device, comprising a vine body and a pressurization chamber. The fabrication of both components is complex due to the multiple roles each component must play in the device's operation.

Vine The vine is constructed using a 2.5 mil polyolefin lay-flat tube, which is adjusted to a width of 31.5 mm and cut into a 750 mm long strip using a heat sealer (eBay, USA). This strip is then loaded onto a helical mold with a pitch height of approximately 2 mm. The 2 mm spacing is specifically reserved to accommodate the thickness of the stabiliztion mechanisms integrated into the device.
Material selection plays a crucial role in the outcome of the preform. While Low-Density Polyethylene (LDPE) is commonly used for vine robots, its conformity under heat treatment is suboptimal. After extensive evaluation, polyolefin (POF) was chosen for its high heat-shrink ratio and excellent conformity to underlying shapes when heat treated. This makes it ideal for maintaining the vine's precise dimensions and ensures that the material retains its shape well under pressure.
Before shrinking, the vine has a circumference of 63 mm. After heat treatment, the vine shrinks to a circumference of 40.83 mm, giving the polyolefin (POF) a shrink ratio of approximately 1.54. To achieve such a high curvature in the vine, the heat gun needs to be positioned mostly on the interior of the vine. This technique ensures that the inner radius of the vine conforms to the helical mold during the shrinking process.
Once positioned on the mold, a heat gun (Makita Australia, Australia) is used to shrink the polyolefin material to conform to the mold's helical shape at a temperature of 105 degrees Celsius. The temperature is a critical factor in forming the preprogrammed shape of the vine. If the temperature is too high, it can cause the material to burn, leading to holes in the vine and compromising its growth performance. Conversely, if the temperature is too low, the material may not shrink sufficiently to conform to the mold, resulting in an inaccurate shape. The appropriate shrinking temperature varies depending on the material and its thickness, so it must be experimentally determined for each application.
Following the shrinking process, the vine is unloaded from the helical mold by rotating the inner mold out after the heat-shrinking process. The uniformity of the mold shape facilitates this demolding and is

a key reason for the helical configuration—it simplifies both growth and fabrication. Once unmolded, the TGS retains its programmed helical shape due to the properties of polyolefin, a compliant yet sufficiently rigid material that behaves similarly to a thin plastic film. This rigidity allows the TGS to maintain its shape when no external force is applied. The TGS then undergoes a thorough quality check to ensure its hermeticity. The vine must be able to hold pressure with continuous airflow input; otherwise, it is deemed unsuitable for use. If the vine cannot maintain pressure, it will not allow proper growth during deployment, compromising the device's functionality. Ensuring the vine's ability to hold pressure is critical, as any leakage would prevent it from effectively serving as a cardiac assistive device.

Pressurization chamber The growth of the vine requires a controlled pressure build-up. Given the additional load from the muscle that the device must drag along during deployment, a deployment chamber is essential. This chamber serves to store the everted material, including the vine and the muscle, ensuring that the device can be deployed efficiently and safely.
The deployment chamber is custom-made using polycarbonate tubes and sheets. Polycarbonate was selected due to its superior mechanical properties, particularly its malleability under stress. Unlike acrylics, which can shatter when they fail, polycarbonate deforms, making it a safer option for applications requiring pressure containment. Although the pressures involved in the deployment process are relatively low, far below the maximum capacity of the chamber, safety considerations are always a priority. A failed chamber, even under low pressure, can result in a generic explosion that poses a risk during experiments.
The design of the chamber draws inspiration from pipe flange connections. The assembly uses bolts and nuts to secure two sheets with the polycarbonate tube placed between them. A custom gasket, made from Smooth-Sil 960 (Smooth-On Inc.), is used to seal the chamber. This gasket not only prevents air leakage but also helps secure the placement of the tube by providing an indented groove where the tube sits. When the bolts are tightened around the two flat sheets, the chamber is sealed, allowing the necessary pressure to build up for vine growth.
The chamber is connected to an air compressor through air fitting parts, enabling direct control of the internal pressure via a pressure regulator. This setup ensures that the pressure within the chamber can be precisely managed, which is critical for the controlled deployment of the vine.
The deployment chamber undergoes rigorous quality checks to ensure it meets two primary criteria: volume capacity and sealing integrity. The first aspect of quality checking focuses on the chamber's ability to accommodate the everted vine and muscle. It must have a sufficiently large volume to store these components during the deployment process without causing any constraints or interference. This capacity is crucial for the proper functioning of the device, as inadequate space could hinder the smooth deployment of the vine and muscle.
The second and most critical aspect of quality checking is ensuring the chamber's sealing integrity. The chamber must be completely airtight to allow for the necessary pressure build-up when connected to the air circuit. Any leaks in the chamber could fail to achieve the required pressure, compromising the device's performance and potentially leading to unsuccessful deployment. To verify sealing integrity, the chamber is subjected to thorough tests under controlled conditions, ensuring it can maintain pressure over a sustained period with minimal loss.

Feeder Relying solely on the propulsion force of the vine to drag along three water-filled muscles can be challenging. Also, this approach may not provide sufficient control to move the muscles effectively, especially in delicate operations like surgical procedures. To overcome this limitation, a feeder mechanism is incorporated to guide the muscles into the vine. This feeder ensures that the muscles are moved forward efficiently and at a controlled pace, which is crucial for precise device deployment. Controlling the growth speed of the vine is essential for surgeons, as it allows them to deploy the device at a rate they are comfortable with, ensuring a safer and more controlled procedure.
The feeder mechanism consists of three main components. The first is the outer case, which stabilizes the position of the other components and maintains the structural integrity of the feeder. The second component is a hand crank that extends outside the pressure chamber, allowing for manual control. This

feature is vital for making precise adjustments to the feeding rate during operation. The third component is a set of dual drive gears, which function similarly to a 3D printer extruder. These gears grip the muscles tightly and feed them forward as the crank is rotated, ensuring a smooth and controlled feeding process.

Each component of the feeder is customized based on the geometry of the muscles and the thickness of the vines to ensure compatibility and optimal performance. The case acts as a framework for the gears and crank and is taped to the upper lid of the pressure chamber. This setup ensures that the feeder muscles are directly fed into the cavity of the vine. The dual drive gears are particularly critical, with a pitch diameter of 16.5mm. These gears are 3D printed with PLA, with layers oriented perpendicular to the direction of force to better distribute the load. The upper part of the dual drive gear, which grips the muscle, is SLA printed. This printing method provides higher precision and is better suited for delicate parts, where FDM printing may not achieve the required quality. The two parts of the gear are joined with super glue, and a heat shrink layer is added to provide additional tolerance, acting as a buffer between the muscle and the rigid gear. This layer helps prevent permanent deformation of the artificial muscles and ensures a more secure grip. The hand crank and the driving gear are mechanically coupled through an indent in the gear design, ensuring that all rotational force applied to the crank is efficiently transferred to the gear. To prevent failure due to stress concentration, the SLA-printed crank is reinforced with a steel rod in areas prone to stress. This coupling ensures that the feeding process is fully controlled and reliable.

The primary quality check for the feeder mechanism focuses on its ability to grip and feed the muscles effectively. A loose grip can lead to reduced control, compromising the precision required for surgical applications. Ensuring a secure and reliable grip is essential for the feeder to function as intended, maintaining controlled deployment of the vine and muscles. By testing the feeder's performance under various conditions, potential issues can be identified and addressed, ensuring the mechanism operates reliably and safely in its intended application.

S2.3 Stabalization Mechanism

The stabilization mechanism is a critical component of the device, and its effectiveness is crucial to the device's overall success. Currently, the device utilizes commercially available materials rather than a customized stabilization mechanism, as it is still in the early stages of development. The fixation mechanism must address five key challenges: 1) it must be strong enough to withstand the forces required during device operation, 2) it must allow for reversible fixation to enable safe device removal, 3) the hydrophobic nature of the POF makes attachment difficult, 4) the device must be able to complete the attachment process as it grows, and 5) the attachment mechanism must not impede the vine's growth motion.

Given these challenges, conventional biocompatible adhesives are unsuitable. Mechanical attachment methods, such as hook-and-loop fasteners (like Velcro) and dual lock mechanisms, have proven to be ideal for this application. These options provide high shear strength, which is particularly important for securely attaching the device around the aorta. Velcro can support approximately 199.13 PSI per square centimetre, while Dual Lock can withstand 72 PSI per square centimetre. This means that even a relatively small area of these attachment mechanisms is sufficient to secure the device effectively. Additionally, their flexibility and compact size minimally hinder the eversion of the vine, ensuring they do not obstruct its growth and facilitating automated device deployment.

Furthermore, the hydrophobic nature of the vine material poses a challenge for chemical bonding, as it would require the use of strong and potentially hazardous chemicals. The safer alternative is to use physical bonds to attach the stabilization mechanisms to the vine. The smooth surface and strength of the POF provide an ideal base for applying stabilization tape. These small tapes are strategically placed around the vine in an alternating pattern, ensuring they do not coalesce at the eversion tip, which could obstruct vine growth. The stabilization mechanisms are confined to the front section of the vine, making reinforcement of the stabilization straightforward since all components are easily accessible. Access to these areas is straightforward due to the ministernotomy, which provides direct access to the aorta.

S2.4 Contraction

The contraction unit utilizes the Hydraulic Filament Artificial Muscle (HFAM), developed by Phan et al. [11], to achieve contraction. HFAM functions the same way as an inverse pneumatic artificial muscle that elongates when pressurized and contracts upon pressure release. For this device, the muscle operates at an elongation of 60% - 100% range.
For detailed fabrication steps, refer to Phan et al's article[11]. Briefly, the muscle is constructed by placing a 290 mm rubber tube within a metal spring coil stretched by 5%, resulting in a rest coil length of 27.55 mm. The rubber tube is filled with a hydraulic medium, which, being incompressible, ensures that any increase in fluid volume directly translates to muscle elongation. One end of the muscle is secured for free movement, while the other is attached to a fluid transmission tube and reservoir. As fluid is added using a linear actuator, pressure builds up. The metal coil constrains radial expansion, causing the muscle to elongate axially. When the fluid volume is withdrawn, the stored potential energy is released, resulting in rapid muscle contraction.
To compress against 120 mmHg, three artificial muscles are required. The rationale for using three muscles is detailed in the subsequent calculation section. These calculations demonstrate that incorporating three muscles ensures the device can reliably displace the required volume under various conditions, providing an added safety margin to account for potential fluctuations in performance or operating conditions. These muscles are connected at the fluid transmission tube using T connectors to ensure even pressure distribution, which is crucial for maintaining uniform elongation across all three muscles when pressurized. This uniform elongation ensures that the contraction and relaxation forces applied to the aorta are consistent. One end of each muscle is attached to the vine's tip, while the other end is secured to the base with a 3D-printed end cap. This configuration allows the contraction and relaxation of the muscles to translate into changes in the device radius, effectively compressing and relaxing the ascending aorta.

Maximum Force Calculation The calculation presented here is done under simplified conditions. Based on the outcome achieved, we can confidently conclude that it provides a fundamental starting point. The simplifications made include 1) The force applied is distributed evenly over the entire active region of the device. 2)The active area remains constant during the device's operation. To justify the second assumption, although the radius of the underlying structure decreases upon compression, it does not vanish. Instead, the compressed regions fold onto themselves, ensuring that the total active area remains constant.
To perform the calculation, we utilize a straightforward Pascal pressure relationship, where the correlation between force and pressure is expressed as:

$$P = \frac{F}{A}.$$

Given that the device is designed with a helical pitch of 13 mm, the longitudinal length of a fully deployed device is:

$$13\,\text{mm} \times 4.5\,\text{turns} = 58.5\,\text{mm}.$$

With a target pressure of 120 mmHg, equivalent to 16000 Pa, the radial force requirement is calculated using the formula:

$$\text{Targeted Pressure} = \frac{F}{\text{Length} \cdot \pi \cdot \text{Aortic Diameter}}.$$

Substituting the values:

$$16000 = \frac{F}{58.5 \times 10^{-3} \cdot \pi \cdot 29 \times 10^{-3}}.$$

The required radial force is calculated to be approximately 85.27 N. Comparing this value to the maximum force output of a single artificial muscle at 80% elongation (∼ 40 N), it is evident that three artificial muscle fibers can collectively provide the required force to perform counterpulsation on the ascending aorta. This configuration offers a safety margin of around 30 N to account for potential force losses during dynamic conditions.

S3 Illustrative Implantation Guide

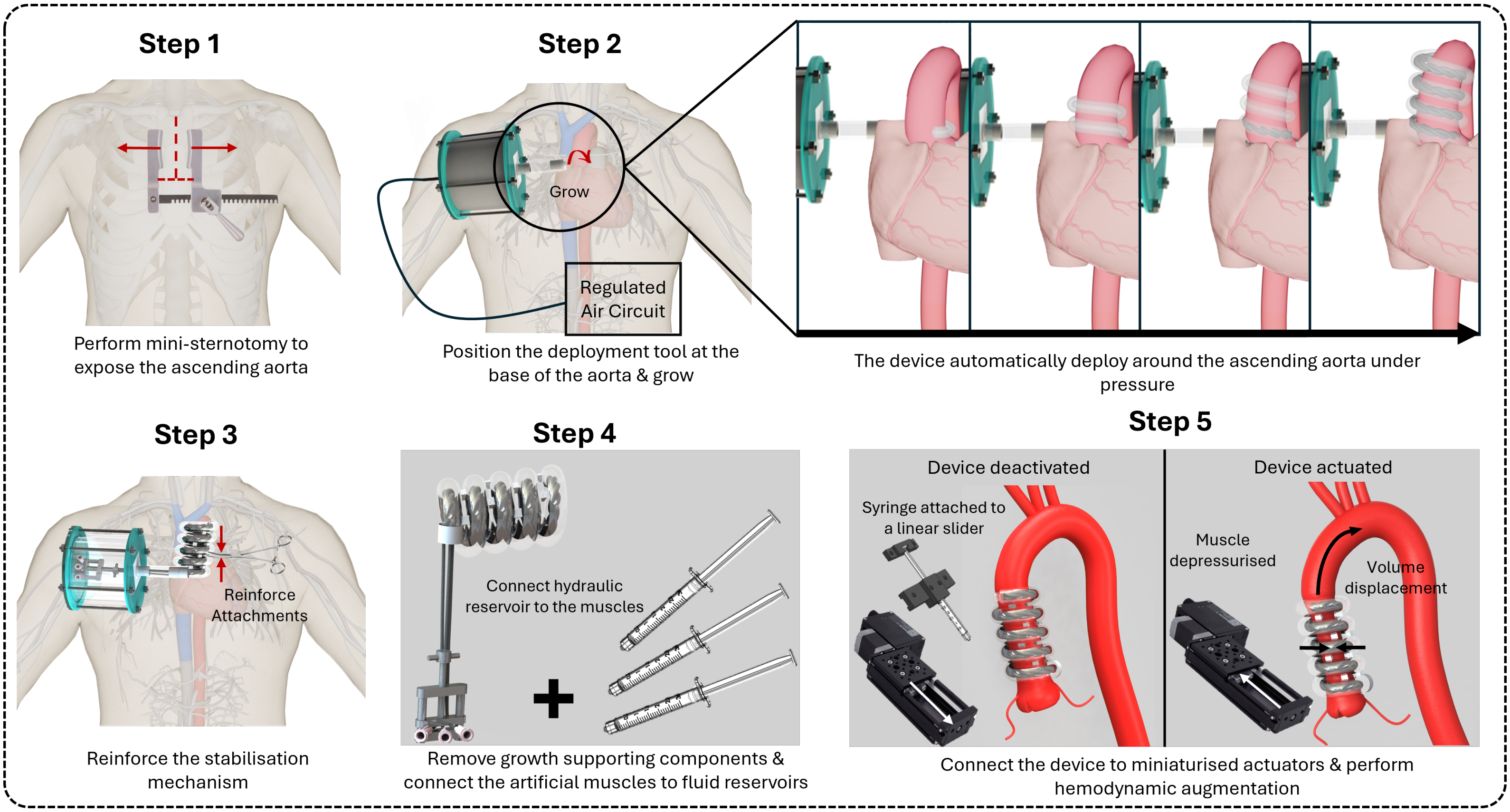

Figure S1: Deployment Process of the Device for Hemodynamic Augmentation.

As illustrated in **Figure 1b**, the deployment process begins with a mini-sternotomy to expose and clear the connective tissues surrounding the ascending aorta (Step 1). The Da Vinci surgical robot is utilized during this step, providing precise dissection and enhanced visualization of the aorta through its integrated camera system. This ensures accuracy and reduces risks associated with tissue preparation.
The device leverages the EACD's ability for controlled self-deployment around the aorta, offering the potential for a minimally invasive implantation. While the initial in vivo studies may necessitate a more invasive surgical procedure, such as a full midline sternotomy, to establish a systematic implantation protocol, advancements in clinical experience and implantation techniques could pave the way for more minimally invasive approaches. The reduced invasiveness of this method is enabled by the device's unique deployment mechanism, a specialized soft robotic system that extends via tip eversion[12], [13], [14]. This mechanism allows the robot to navigate complex anatomical environments with precision.
The deployment relies on an external pressure chamber that houses the everted TGS material and the pressurized hydraulic muscles. This chamber is used exclusively during deployment and is removed once the process is complete. As pressure builds within the chamber, the TGS extends into its preformed helical configuration, encapsulating the aorta. Shape programming plays a critical role at this stage, ensuring precise geometry while minimizing interaction with surrounding tissues. During deployment, the artificial muscles remain elongated, with their elongation maintained by pressure held within the trans-

mission tubes using Hoffman clamps. The device is designed to wrap around the largest diameter of the aorta during the cardiac cycle, reducing the risk of compressive forces during deployment and minimizing potential surgical challenges.

Once the device is positioned, it is stabilized using a flexible and reversible mechanism (Step 3). Initially, the vine's helical shape is maintained by internal pressure, allowing the stabilization mechanism to be securely reinforced. This stabilization mechanism is crucial for locking the artificial muscles into their designed configuration, enabling the conversion of axial contraction into radial constriction of the aorta during operation. This configuration facilitates effective counterpulsation therapy. The reversibility of the stabilization mechanism also simplifies device removal, ensuring the procedure remains minimally invasive. Once stabilized, the pressure chamber is removed, and all internal pressure within the TGS is released passively from the compression of surrounding tissue. At this stage, the stabilization mechanism alone preserves the structural integrity of the device, with the TGS serving as a scaffold to maintain the helical shape.

Following stabilization, the hydraulic muscles are activated. These hydraulically driven artificial muscles, developed by our team [11], mimic the behavior of natural muscle filaments. It elongates when pressurized and contracts when pressure is released. During deployment, the muscles are disconnected from their actuation sources to ensure the sealing of the pressure chamber. After all deployment-related components are removed (Step 4), the muscles are reconnected to their fluid reservoirs and linked to linear actuators, preparing them for operational use. To ensure safety, the Hoffman clamps are only removed after the linear actuators are in place, ensuring the device maintains its maximum diameter until the control system is ready. During operation, the muscles contract radially around the aorta, displacing volume and augmenting aortic pressure, thereby unloading the ventricles and providing hemodynamic support.

S4 Empirical Modeling

Table S3: Assumptions for the Actuator Model

**Assumption**	**Description**
Quasi-Hydrostatic State	The system operates in a quasi-hydrostatic state, ensuring that the fluid's momentum does not affect the actuator's response. This assumption helps in focusing solely on the static pressure distribution.
Uniform Pressure Distribution	The pressure is evenly distributed on the inner surface of the rubber tube, resulting in uniform deformation. This ensures that the deformation analysis remains consistent throughout the tube.
Poisson's Ratio	The Poisson's ratio of the rubber tube is assumed to be 0.5. This implies that any change in the tube's diameter is fully converted to a change in its length, simplifying the deformation calculations.
Radial Expansion Constraint	The spring coil restricts radial expansion. This means that the expansion force is redirected, influencing the axial deformation of the actuator.
Symmetrical Displacement	The muscle's forward and backward displacements are assumed to be identical, preventing displacement accumulation. This maintains the system's equilibrium and simplifies the dynamic analysis.

Table S4: Variable Descriptions

**Variables**	**Descriptions**
F_{out}	Total output force of the muscle. This is the sum of all individual forces produced by the muscle.
F_{axial}	Output force produced by the muscle in the axial direction.
$F_{torsional}$	Output force produced by the muscle that results in the torsion of the artificial muscle.
F_{radial}	Radial force the muscle produces when arranged in a helical configuration.
E	Elastic modulus of the inner rubber tube.
r_o	Outter diameter of the rubber tube.
r_i	Inner diameter of the rubber tube.
K_c	Spring constant of the outermost containing coil.
l_o	Initial length of the artificial muscle.
x	Displacement of the artificial muscle.
ϵ	$\frac{x}{l_o}$ Strain of the of the artificial muscle.
V_{in}	Input volume from the driving source.
P_m	Hydraulic pressure within the artificial muscle.
K_r	Spring constant of the rubber tube is a function of the input volume.
$A_(ro)$	$\pi(r_o^2 - r_i^2)$ Cross sectional area of the rubber tube.

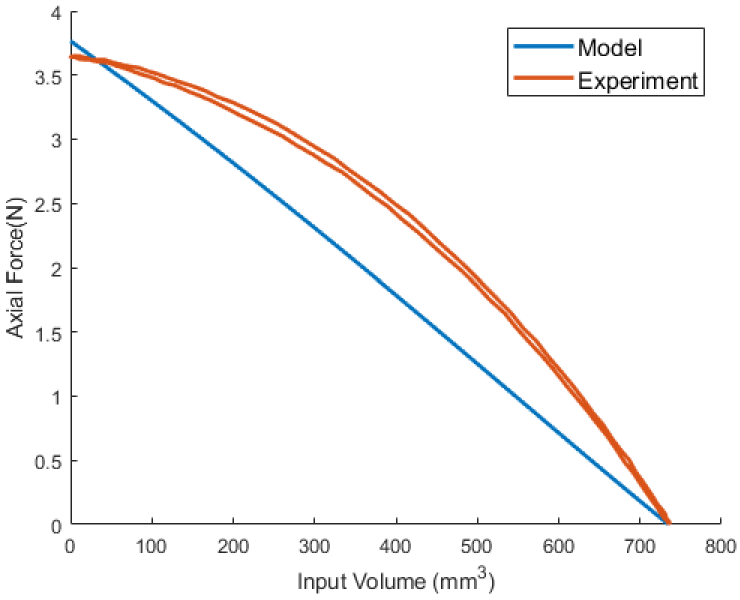

Figure S2: Model and experimental axial force

S5 Device Integration Test

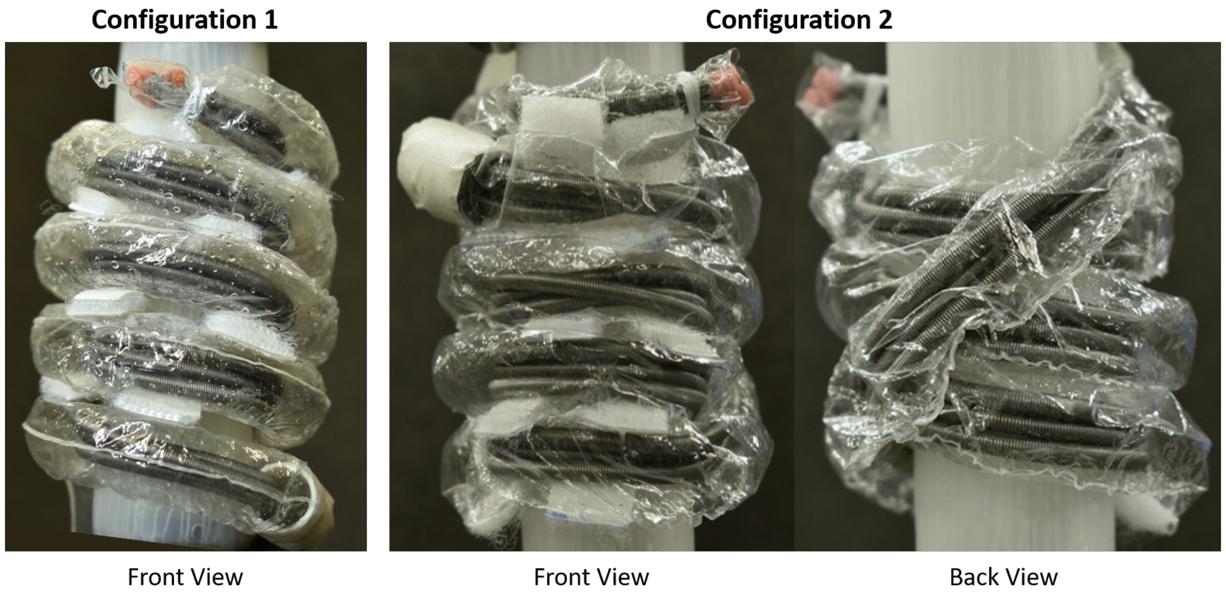

Figure S3: Photograph of the device prototype configuration 1 and 2

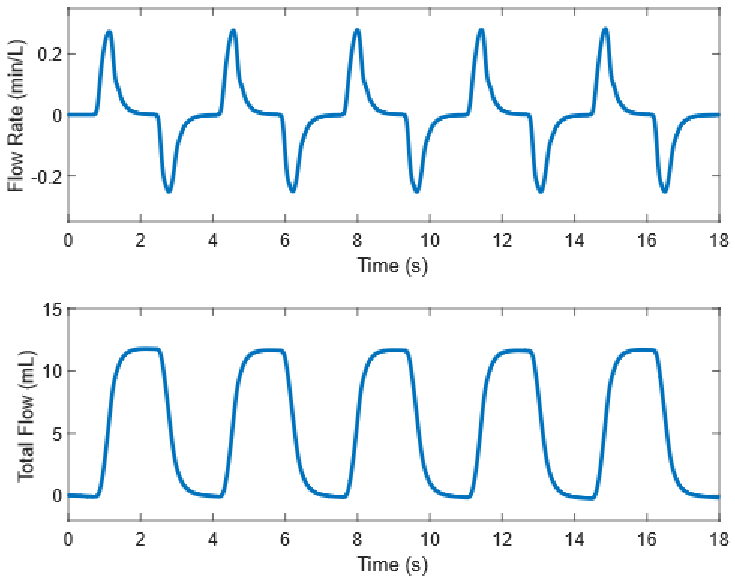

Figure S4: Exemplar device output flow patterns

1 # S6 *In Vitro* Test Setup Illustrtaion

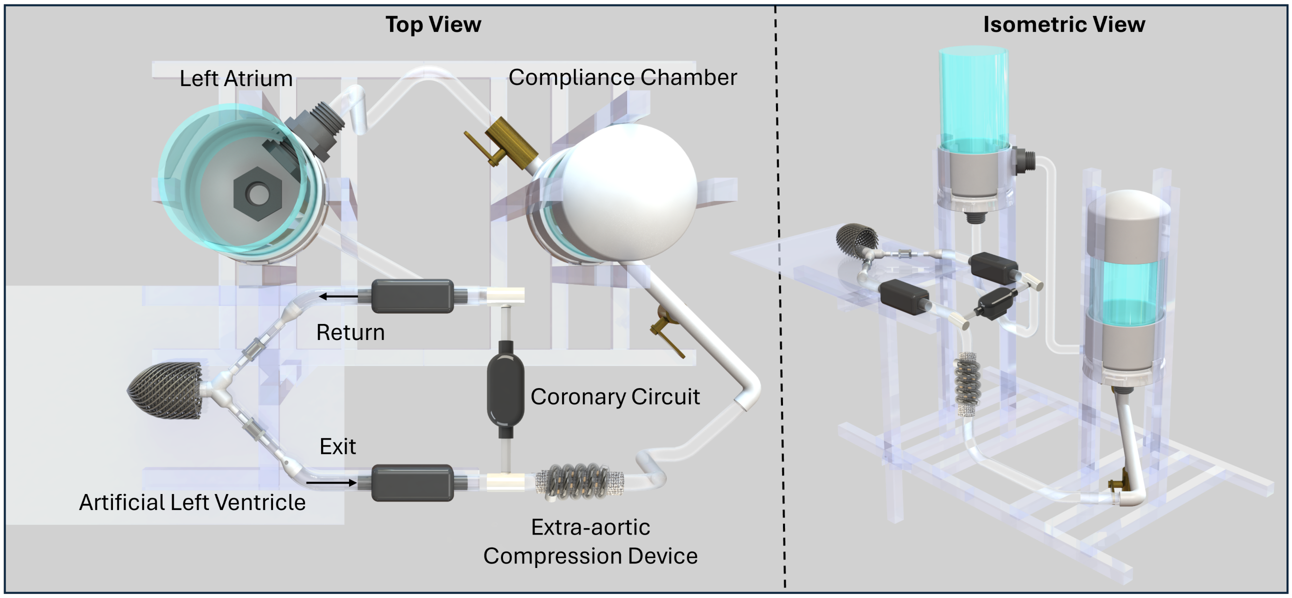

Figure S5: CAD model of the in vitro experimental setup in top and isometric view

2 # S7 Modelled PV at 1BPM and 60BPM

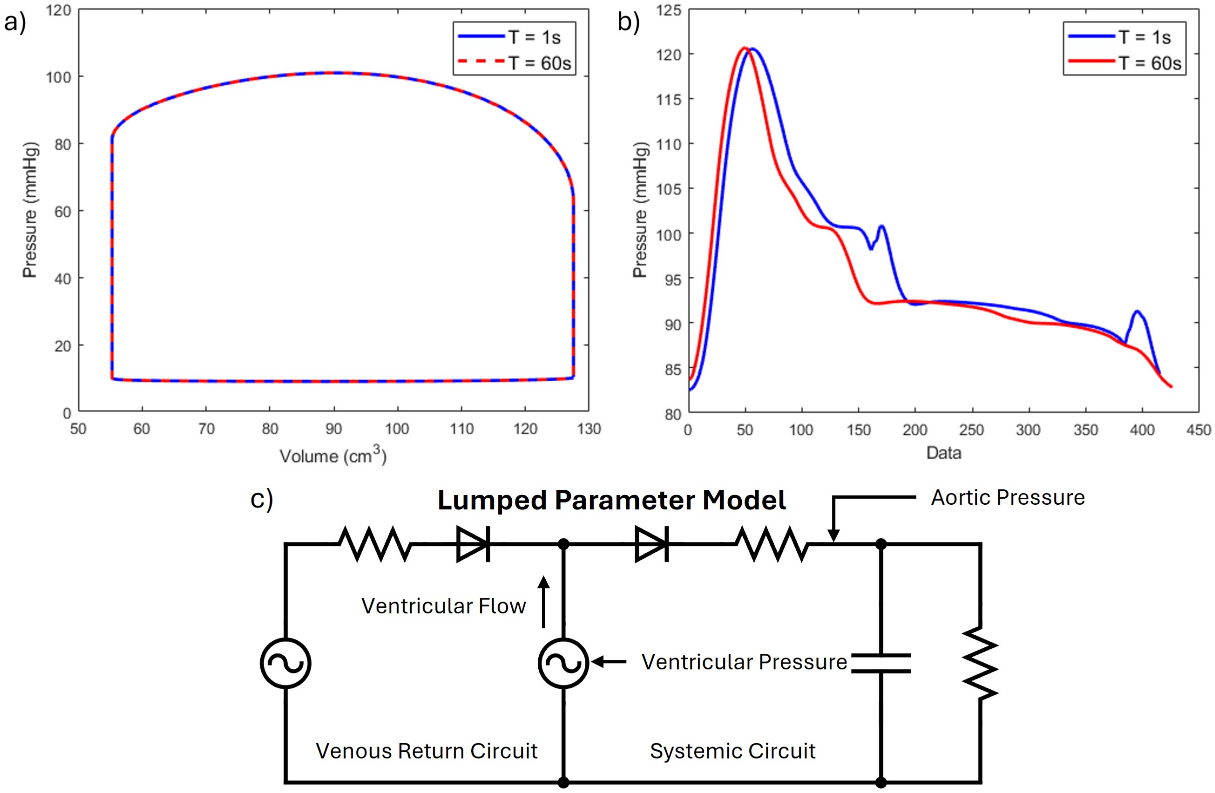

Figure S6: (a) Modelling of the PV loop at 1bpm and 60bpm with proportionally scaled flow parameters. (b)The simulated aortic pressure at the two heart rates. (c) A visual representation of the lumped parameter model used to simulate the EACD test platform, inspired by [15].

S8 *In Vitro* Test Actuation Profile and Supplementary Results

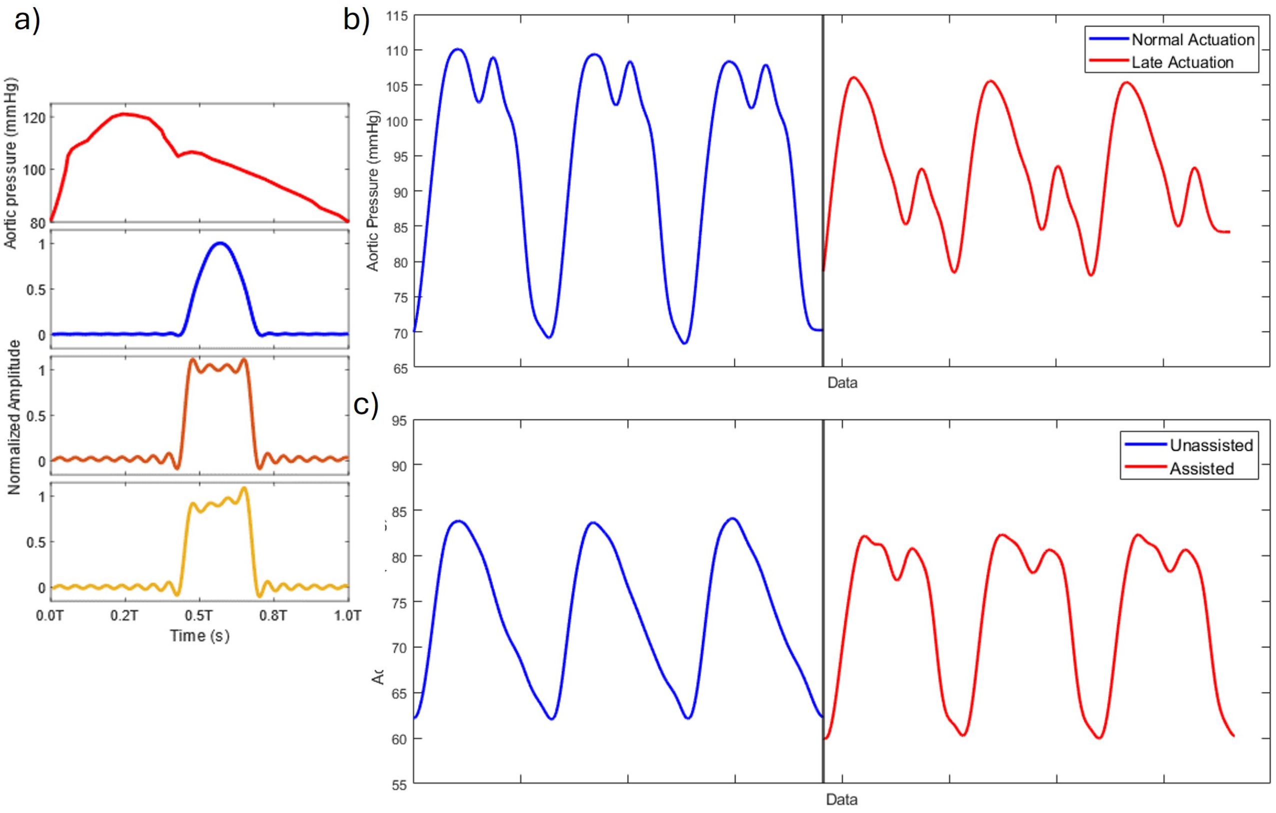

Figure S7: (a) Device actuation input waveform in alignment with the aortic pressure signals. (b) Comparison between device-assisted and unassisted aortic pressure in a less stenotic test. (c) Reference assisted aortic pressure and late actuation aortic pressure.

S9 Coronary Flow Increase

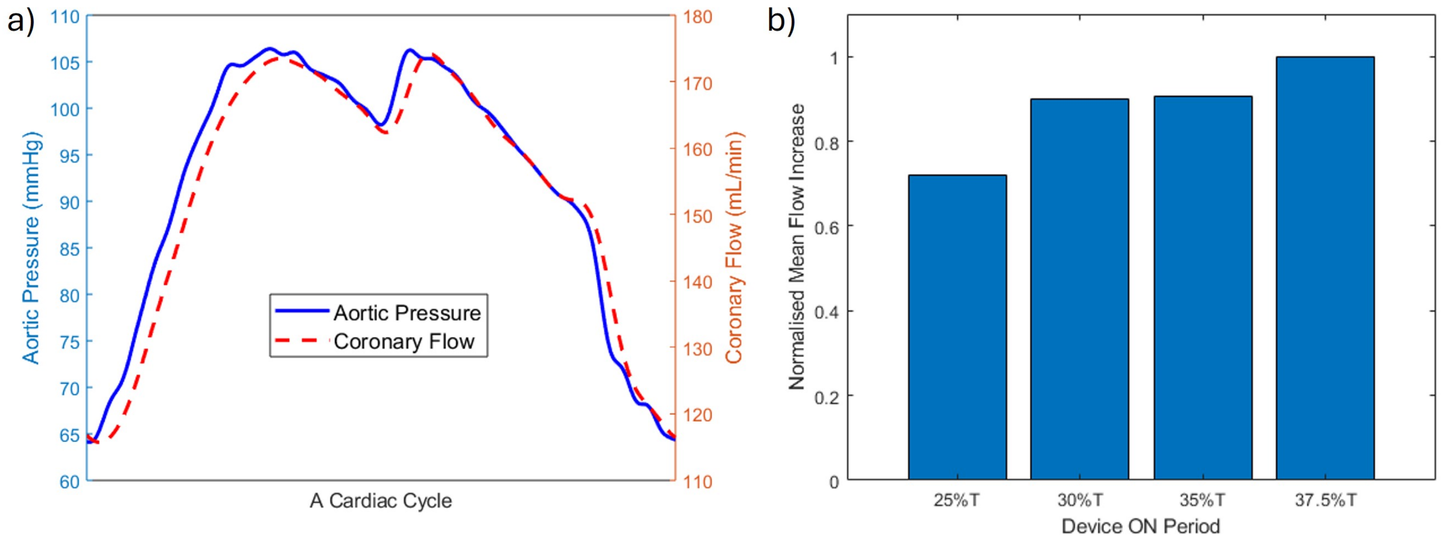

Figure S8: (a) Aortic pressure-driven coronary flow profile under assisted conditions. (b) Normalized increase in coronary flow at different device activation durations.

S10 Preliminary Durability Test

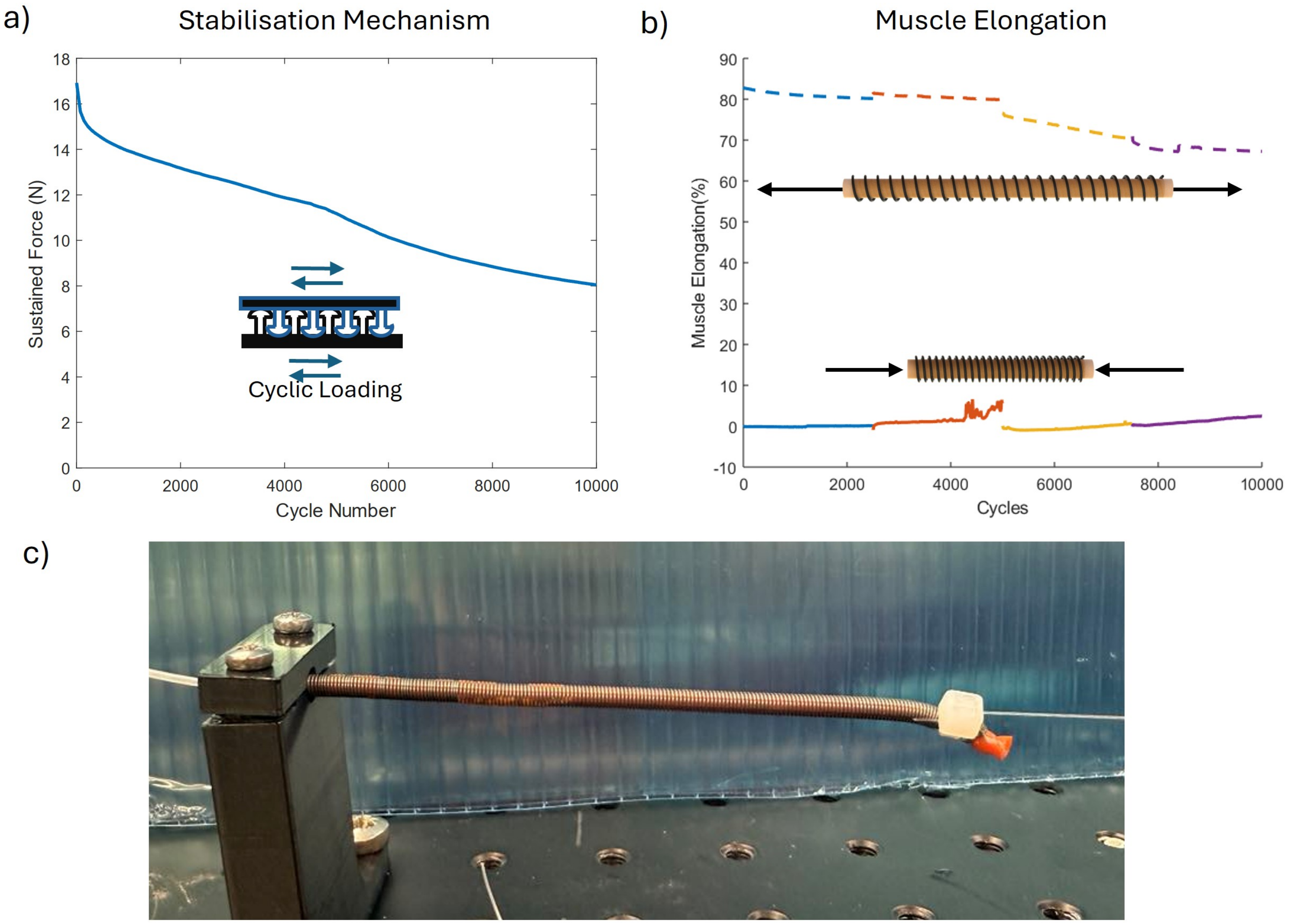

Figure S9: (a) Maximum sustained force of the stabilization mechanism over 10,000 cycles. (b) Elongation and contraction lengths of the muscle filament over 10,000 cycles. (c) Wear condition of the muscle filament after 10,000 cycles.

To evaluate the durability of the device, key components of the EACD were subjected to cyclic loading. **Figure S9a** illustrates the drop in maximum force sustained at the same magnitude of pull over 10,000 cycles. A turning point is observed around 5,000 cycles, marking the onset of detachment from the underlying POF-covered plastic board. The rigidity of the dual-lock tape substrate creates stress concentration points, rather than evenly distributing stress, which leads to delamination of the physically bonded adhesive layer and hence detachment from the flexible TGS beneath.

Figure S9b shows changes in the elongation of the muscle under constant input volume. The muscle was initially targeted to achieve 80% elongation, reflecting its operating conditions. The upper data line represents the muscle's elongation, while the bottom line corresponds to its contraction, with the 0% elongation baseline indicating the fully contracted length of the muscles. Data were recorded in batches of 2,500 cycles to avoid computer crashes during prolonged data acquisition. Over 10,000 cycles, the muscle's elongation capacity decreases, and it is unable to contract to its original length fully. This behavior is attributed to wear, as illustrated in Figure S9c, where the worn outer surface of the rubber tube becomes lodged between the spring coils, preventing full spring contractions. Additionally, the disintegration of the rubber tube impacts the elongation capability of the muscles. At the end of 10,000 cycles, the contracted length increased by 2.44%, while the maximum elongation decreased from approximately 80% to 67.2%. However, no signs of failure were observed in other critical areas, such as junctions or attachment points.

During the second batch of the muscle elongation test, the attachment of the muscle to the testing jig came loose, resulting in fluctuations observed between 4,000 and 5,000 cycles. This issue is unrelated to the muscle itself and does not indicate material failure.

S11 Experimental Setup

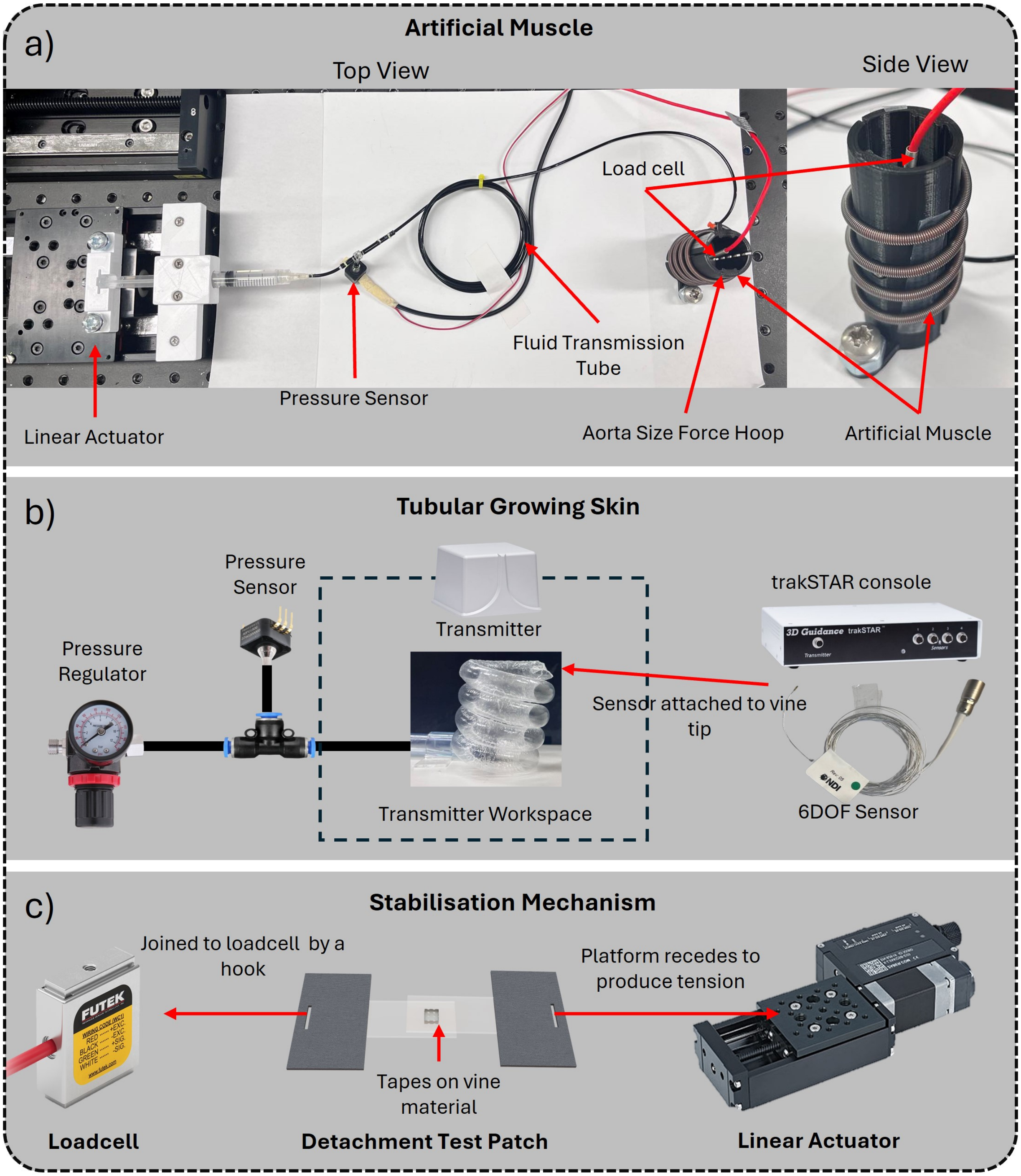

Figure S10: Illustration of the experimental setups for modular test of the device

References

[1] H. Parissis, V. Graham, S. Lampridis, M. Lau, G. Hooks, P. C. Mhandu, *Journal of Cardiothoracic Surgery* **2016**, *11*.

[2] C. D. Kontogiannis, K. Malliaras, C. J. Kapelios, J. W. Mason, J. N. Nanas, *World Journal of Transplantation* **2016**, *6* 115.

[3] M. Krishna, K. Zacharowski, *The Internet Journal of Thoracic and Cardiovascular Surgery* **1999**, *2*, 2.

[4] V. Jeevanandam, D. Jayakar, A. S. Anderson, S. Martin, W. Piccione, A. Heroux, J. Wynne, L. W. Stephenson, J. Hsu, P. S. Freed, A. Kantrowitz, *Circulation* **2002**, *106*.

[5] C. S. Hayward, W. S. Peters, A. F. Merry, P. N. Ruygrok, P. Jansz, G. O'Driscoll, R. I. Larbalestier, J. A. Smith, B. Ho, M. E. Legget, F. P. Milsom, *The Journal of Heart and Lung Transplantation* **2010**, *29* 1427.

[6] V. L. Sales, P. M. McCarthy, *Current Heart Failure Reports* **2010**, *7* 27.

[7] M. E. Legget, W. S. Peters, F. P. Milsom, J. S. Clark, T. M. West, R. L. French, A. F. Merry, *Circulation* **2005**, *112*, 9_supplement.

[8] I. Pirozzi, A. Kight, X. Liang, A. K. Han, D. B. Ennis, W. Hiesinger, S. A. Dual, M. R. Cutkosky, *Advanced Materials Technologies* **2022**, *8* 2201196.

[9] C. T. Starck, J. Becker, R. Fuhrer, S. Sündermann, J. W. Stark, V. Falk, *Interactive Cardiovascular and Thoracic Surgery* **2013**, *18*, 1 13–16.

[10] M. Almanza, F. Clavica, J. Chavanne, D. Moser, D. Obrist, T. Carrel, Y. Civet, Y. Perriard, *Advanced Science* **2021**, *8* 2001974.

[11] P. T. Phan, M. T. Thai, T. T. Hoang, N. H. Lovell, T. Nho Do, *IEEE Access* **2020**, *8* 226637.

[12] E. W. Hawkes, L. H. Blumenschein, J. D. Greer, A. M. Okamura, *Science Robotics* **2017**, *2* eaan3028.

[13] J. D. Greer, L. H. Blumenschein, R. Alterovitz, E. W. Hawkes, A. M. Okamura, *The International Journal of Robotics Research* **2020**, *39* 1724.

[14] L. H. Blumenschein, M. M. Coad, D. A. Haggerty, A. M. Okamura, E. W. Hawkes, *Frontiers in Robotics and AI* **2020**, *7*.

[15] S. Dokos, *Modelling organs, tissues, cells and devices : using MATLAB and COMSOL multiphysics*, Springer, Berlin, Germany, **2017**.
